# Highly sensitive AIEE active fluorescent probe for detection of deferasirox: extensive experimental and theoretical studies†

**DOI:** 10.1039/d4ra03548h

**Published:** 2024-07-08

**Authors:** Kainat Khurshid, Sohail Anjum Shahzad, Mohammed A. Assiri, Alam Shabbir, Tayyeba Javid, Hasher Irshad

**Affiliations:** a Department of Chemistry, COMSATS University Islamabad, Abbottabad Campus, University Road Abbottabad 22060 Pakistan sashahzad@cuiatd.edu.pk; b Department of Chemistry, Faculty of Science, King Khalid University P.O. Box 9004 Abha 61413 Saudi Arabia; c Research Centre for Advanced Materials Science (RCAMS), King Khalid University P. O. Box 9004 Abha 61514 Saudi Arabia

## Abstract

High concentrations of deferasirox (DFX) in living organisms cause hepatic, gastric and renal malfunctions. Therefore, it is significant to establish an accurate and efficient approach for the detection of deferasirox (DFX) to protect public health. Herein, we synthesized a thiourea-based diphenylacetamide probe MPT for the effective sensing of deferasirox through the fluorescence quenching phenomenon. The designed probe MPT shows a fluorescence quenching response toward deferasirox (DFX) through photo-induced electron transfer (PET). Furthermore, DFT studies were performed to support the experimental results. ^1^H-NMR titration experiment was used to explore the interaction type between probe MPT and DFX. The existence of non-covalent interactions was verified with spectroscopic studies that were assisted by NCI studies, QTAIM and SAPT0 analysis. Dynamic light scattering (DLS) analysis and scanning electron microscopy (SEM) were used to investigate the complexation of probe MPT with DFX. Moreover, the on-site solution phase and solid-state detection of DFX by probe MPT are executed. Additionally, the practical applications of probe MPT to sense DFX were also revealed in human plasma as well as in artificial urine samples.

## Abstract 2

Representation of side effects by overdose of deferasirox (DFX) and sensing studies performed for DFX detection.

## Graphical abstract

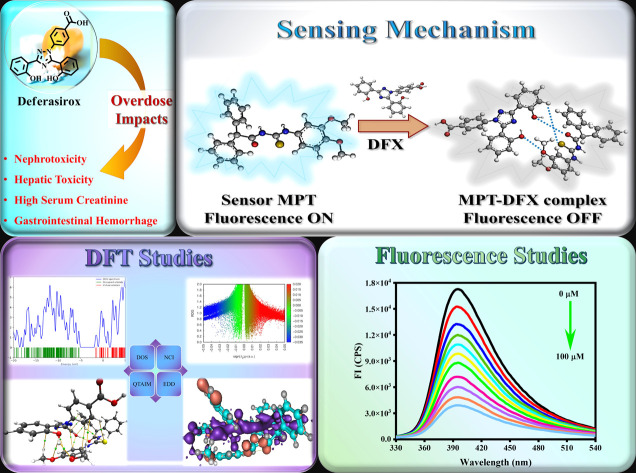

## Introduction

Iron chelators constitute a class of drugs which prevent iron toxicity in patients who require long-term blood transfusion such as those suffering from different types of anaemia and thalassemia. Deferoxamine (DFO), deferasirox (DFX) and deferiprone are some common iron chelators inhibiting iron overload.^1^ Deferasirox, 4-[3,5-bis(2-hydroxyphenyl)-1,2,4-triazol-1-yl] benzoic acid, is a drug administered orally in the dispersible form to treat drug overload in patients having myelodysplastic anaemia, sickle cell anaemia, hemolytic anaemia, aplastic anaemia but most importantly β-thalassemia.^2^ DFX is associated with the non-proliferation of cancer cells as they require more iron for their growth.^3^ DFX demonstrates anti-proliferative activity against breast cancer,^4^ pancreatic cancer,^5^ melanoma and lung cancer.^6^ DFX, one of the best chelators for thalassemia patients, exhibits strong interaction with human serum albumin (HSA) in blood. A hexacoordinated Fe(iii) complex is formed by two molecules of DFX.^7^ However, excess and continuous intake of DFX may cause nephrotoxicity,^8^ gastrointestinal haemorrhage, hepatic toxicity and increased serum creatinine level.^9^ Because of its adverse effects on human health DFX level needs to be monitored and controlled. Some commonly used techniques such as plasma mass spectrometry,^10^ electrocatalytic detection,^11^ voltammetry,^12^ capillary electrophoresis,^13^ atomic absorption spectroscopy^14^ and LC-MS^15^ are employed for the detection of iron chelators. These techniques are known to have many limitations mainly poor selectivity, low sensitivity, complex sample treatment, long time analysis and expensive equipment.^16^ Therefore, the development of new analytical techniques is the prime requirement for selective, inexpensive, sensitive and accurate detection of DFX. Recently, fluorescent probes have emerged as efficient analytical tools because of their simplicity, low detection limit, distinguished selectivity, high efficiency, and fast response time.^17,18^

Numerous organic fluorophores with less π-conjugation or higher conjugation^19^ exhibit strong emission in dilute solution but are prone to excimer or exciplex complex formation^20^ in the solid or aggregated state, because of planar geometry and weak intermolecular forces such as π–π stacking,^21^ causing them to become non-emissive. It is known as aggregation-caused quenching (ACQ),^22,23^ limits the practical use of fluorophores to dilute solutions only and obstructs the formation of portable probes.^24^ Aggregation-induced emission (AIE), the inverse phenomenon of ACQ, first reported by Tang *et al.* in 2001^25^ have many advantages over ACQ. AIE luminogens (AIEgens) have little to no fluorescence in dilute solution but show substantial luminescence while in the concentrated form or when they form aggregates in a solvent of poor solubility such as water.^26,27^ Many fluorescent compounds which are poorly emissive in the solution state, establish remarkably enhanced emission upon aggregation^28^ due to the aggregation-induced emission enhancement (AIEE) property, first discovered by Park *et al.* in 2002.^29^ The molecular interactions such as restricted intramolecular rotation (RIR),^30^ π–π interaction, J-aggregates^31^ and inhibition of excited state intramolecular proton transfer (ESIPT)^32^ are some plausible mechanisms for the AIEE property in fluorophores. Predominant characteristics of AIEE active probes, for instance, large Stokes' shift,^33^ excellent emission, characteristic sensitivity, great stability in polar solvents, resistance to photobleaching,^34^ and high SNR (signal-to-noise ratio) make them superior to ordinary probes hence contributing to their applicability in various areas including bio probes,^35^ molecular logic gates, cell imaging, OLEDs as well as explosives sensing. The significance of AIE and AIEE luminogens intrigued scientists to develop fluorescent probes such as benzimidazole-based,^36^ coumarin-based^37^ and graphene oxide-based probes^38^ for the sensing of deferasirox (DFX).

As an extension of our research^39–46^ to develop new fluorescent probes, an easily accessed MPT probe with thiourea functionality was synthesized. The incorporation of thiourea functionality in fluorophore increased the chances of interacting non-covalently with the analyte in the aqueous state. As anticipated, the designed probe MPT becomes emissive in an aggregated and solid-state and exhibits selective quenching against deferasirox. Furthermore, the sensing mechanism was investigated by fluorescence spectroscopy, UV-vis spectroscopy, Job's plot, ^1^H-NMR titration experiment and DFT analysis.

## Experimental

### Synthetic procedure of *N*-((3,4-dimethoxyphenyl)carbamothioyl)-2,2-diphenylacetamide (MPT)

Diphenylacetic acid (3.0 mmol) and SOCl_2_ (6.0 mmol, 2 equiv.) were added in 5 mL THF and the mixture was refluxed at 66 °C for 8 hours with continuous stirring to form diphenylacetyl chloride. Excess SOCl_2_ was evaporated under vacuum. Dry potassium thiocyanate (3.0 mmol) was mixed in acetone, added to diphenylacetyl chloride, and stirred continuously for 4 hours at 20 °C to produce diphenylacetyl isothiocyanate. 2,3-Dimethoxyaniline (3.0 mmol) was mixed in 5 mL acetone and added to the reaction mixture containing diphenylacetyl isothiocyanate to yield *N*-((3,4-dimethoxyphenyl)carbamothioyl)-2,2-diphenylacetamide. The product formed was recrystallized with methanol and was obtained in 88% yield. ^1^H-NMR (400 MHz, CDCl_3_), *δ* ppm: 12.27 (s, 1*H*, NH), 8.86 (s, 1*H*, NH), 7.39–7.32 (m, 5*H*, ArH), 7.31–7.24 (m, 6*H*, ArH) 7.12 (dd, *J* = 8.6 Hz, 1*H*, ArH), 6.83 (d, *J* = 8.6 Hz, 1*H*, ArH), 5.02 (s, 1*H*, CH), 3.86 (s, 3*H*, CH_3_), 3.84 (s, 3*H*, CH_3_); ^13^C NMR (100 MHz, CDCl_3_), *δ* ppm: 177.6 (C), 173.0 (C), 148.7 (C), 147.66 (C), 136.9 (C), 130.5 (C), 129.1 (4 × C), 128.7 (4 × C), 128.1 (2 × C), 116.1 (C), 110.8 (C), 107.95 (C), 59.5 (C), 55.9 (2 × C).

### Spectrofluorimetric experiment

10 μM in water/THF (9 : 1, v/v) was selected as the optimum concentration of probe MPT for the fluorescent titration experiment. This concentration of probe MPT remained the same for other fluorescence studies. 1000 μM stock solution of potentially detectable drugs including deferasirox (DFX), mefenamic acid (MFA), drotaverine (DV), l-methyl folate (l-MTHF), paracetamol (PCM), ebastine (EBA), doxycycline (DOX) and hydroxyurea (HYD) was prepared in water/THF (9 : 1, v/v). In a 1000 μL cuvette, the gradually varying concentrations of drugs from 10 to 100 μM were scanned to observe the emission spectrum of probe MPT. When the probe MPT was irradiated at the excitation wavelength of 305 nm, the probe showed the maximum emission at 394 nm.

### Pretreatment of plasma

Different concentrations of DFX can be present in the plasma of patients. To check whether the probe MPT could be utilised to sense different concentrations of DFX in plasma the different concentrations of DFX were identified by using the standard addition method. The emission of probe MPT was scanned after adding the known concentration of DFX (0 to 100 μM) to the plasma samples.

### Computational studies

DFT studies were executed by using Gaussian 09 software.^47^ Visualization and analysis were done by using Gauss View 5.0 software.^48^ Using ωB97XD/6-31G (d, p) level of theory,^49^ the geometric studies were done to determine the interaction energies among the probe MPT and analyte. To improve the long-range non-covalent interaction ωB97XD/6-31G (d, p) basis set was preferred instead of other DFT functionals. The given formula, which incorporated a basis set superposition (BSSE) correction, was used to find out the interaction energies.

1 *E*_int_ = *E*_c_ − (*E*_s_ + *E*_a_) + *E*_BSSE_

Here *E*_int_ signifies interaction energy, energy of probe (*E*_s_), energy of analyte (*E*_a_), energy of complex (*E*_c_) and basis set superposition error (*E*_BSSE_) correspondingly. Thermodynamic stability is proportional to the interaction energy, it increases with an increase in interaction energy and *vice versa*.

DFT studies including the frontier molecular orbital (FMO), density of states (DOS) and natural bond orbital (NBO) analysis were carried out to examine the electronic properties of cooperating species. When probe MPT interacts with a specific analyte it forms a new energy level that was described with the help of density of state (DOS). FMO analysis reveals the energy level of HOMO and LUMO and their energy gap. GaussSum^50^ software was used to draw the spectra of the density of state (DOS). NBO and EDD were performed to describe the electron transfer between two interrelating species. NBO provides the numerical values while the EDD provides its graphical representation.

Weak intermolecular forces including van der Waals interaction and steric repulsion among two interacting moieties were studied with the help of non-covalent interaction (NCI) and electron density difference (EDD) analysis. VMD and Multiwfn^51^ software was used for the analysis of these non-covalent interactions. The blue and green spikes of the 2D-RDG (reduced density gradient) and 3D isosurface of probe MPT represent the presence of strong van der Waals attractive interactions and hydrogen bonding present between probe MPT and DFX. In contrast, the red spike of 2D-RDG and red patches of 3D isosurface show the presence of repulsive forces. The 3D isosurfaces and 2D-NCI graphs are assessed to determine the type of attractive and repulsive forces between MPT and DFX. The association between electronic density and RDG is described by the given equation.2
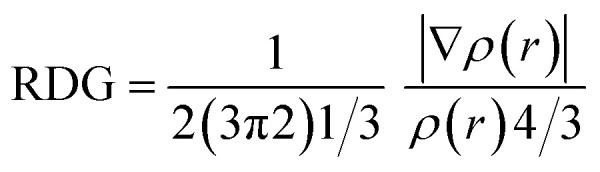
In 3D isosurface, its thickness shows the strength of non-bonding interaction. Hence, the thicker patches denote stronger interaction while spot-like little patches denote weak interactions.

Bader's Quantum theory of atom in molecule (QTAIM) extensively analyzes intermolecular non-covalent interactions.^52^ The topological parameters such as energy density *H*(*r*), potential energy density *V*(*r*), kinetic energy density *G*(*r*) explain the nature of bond while electron density *ρ*(*r*) gives an estimate of the strength of bond critical points (BCPs).

## Results and discussion

### Synthesis

**Scheme 1:**
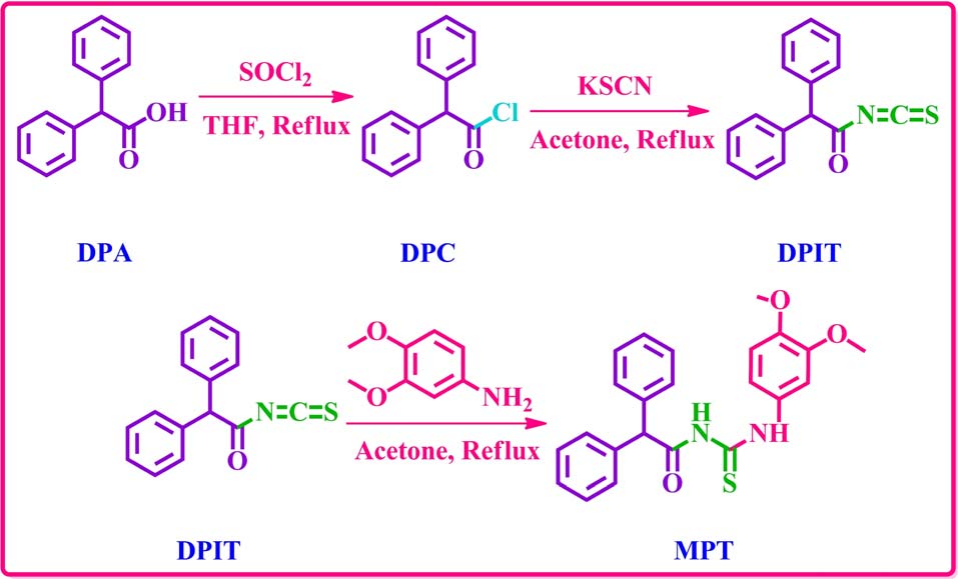
Synthetic strategy to afford the probe MPT.

The desired diphenylacetamide based dimethoxyaniline probe MPT was obtained through the treatment of 3,4-dimethoxyaniline with 2,2-diphenylacetyl isothiocyanate (DPIT) which was obtained by displacement of chloride group from diphenylacetyl chloride (DPC) by thiocyanate upon reaction with potassium thiocyanate (Scheme 1). ^1^H and ^13^C-NMR spectroscopy confirmed the structure of probe MPT. The ^1^H-NMR spectrum substantiated the presence of thiourea functionality by two NH proton signals at *δ* 12.27 ppm and *δ* 8.86 ppm. Moreover, the presence of thiourea moiety is also evident from 177.6 ppm carbon signal in ^13^C-NMR spectrum. The ^1^H, ^13^C-NMR and DEPT-135 spectra of this compound are depicted in the ESI.†

### Optimization and aggregation studies

**Fig. 1:**
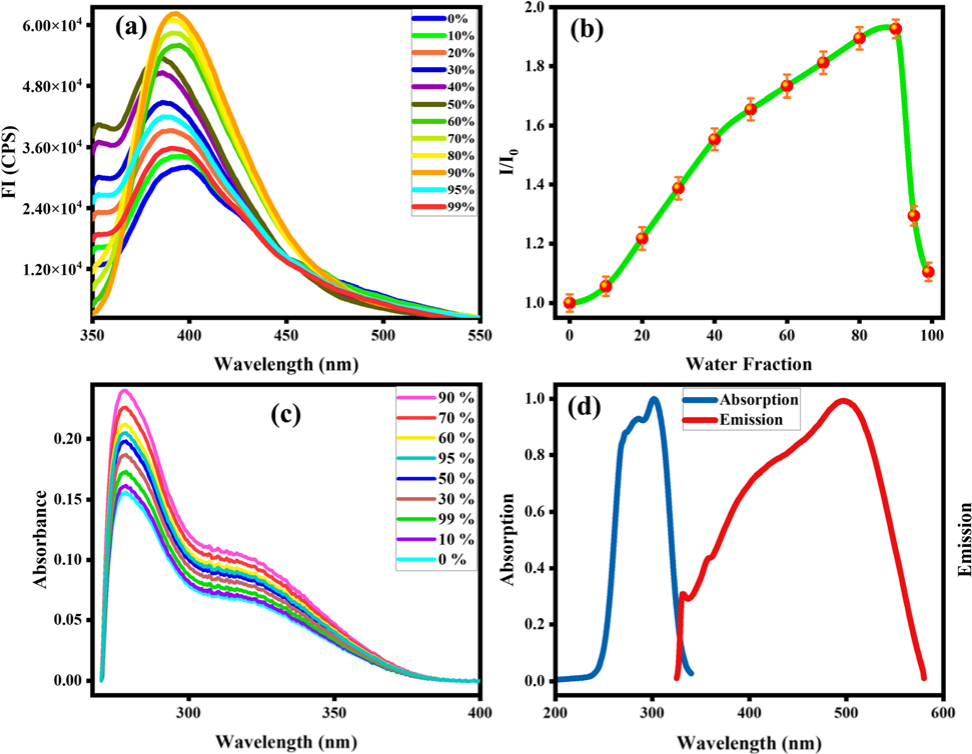
(a) Emission spectra of probe MPT at *λ*_max_ = 384 nm that shifted with increasing water fraction up to 99% at *λ*_max_ = 394 nm, (b) effect of water content on relative emission intensity of probe MPT (c) effect of water fraction on absorbance and (d) absorption and emission spectra of deferasirox.

Organic fluorophores form aggregates with water that tend to alter their fluorescence properties. To observe the consequence of aggregates on the fluorescence of the probe MPT was investigated in THF and water by the gradual accumulation of water fraction (0–90%). The low emission intensity of MPT was observed in pure THF. Surprisingly, probe MPT showed significant enhancement in its fluorescence intensity with the rise in water fraction up to 90% at *λ*_max_ 394 nm that confirm the probe MPT was AIEE active. Aggregates restrict the intermolecular rotation that increases the conjugation of excited electrons which enhances the emission intensity of MPT. The probe MPT contains free rotation along single bonds next to the benzene rings. These intramolecular free rotations cease upon gradual insertion of water fraction (fw). Moreover, aggregates planarize the structure of MPT which enhances the emission of probe MPT. Furthermore, there's a bathochromic shift of 10 nm when the water fraction is increased further from 50%. The emission wavelength is 384 nm at lower water fractions but is shifted to a longer wavelength of 394 nm at 60–90% water fraction (Fig. 1a and b). It could be attributed to a minor formation of J-aggregates at high water fractions due to the alignment of molecules in a restricted geometry such that their dipoles are arranged in a head-to-tail manner. Upon further increasing the water content to 95% and 99% the fluorescence intensity was greatly quenched which could plausibly be attributed to excimer formation and concentration quenching. UV absorption spectrum also shows an increase in absorbance upon aggregation, however, absorbance is also decreased at a very high water fraction (Fig. 1c). Resultantly, the 90% water fraction is selected for further fluorescent studies as it gives maximum emission at a comparatively longer wavelength. Quantum yield of MPT in different water fraction was also calculated and reported in Table S1a.†

**Fig. 2:**
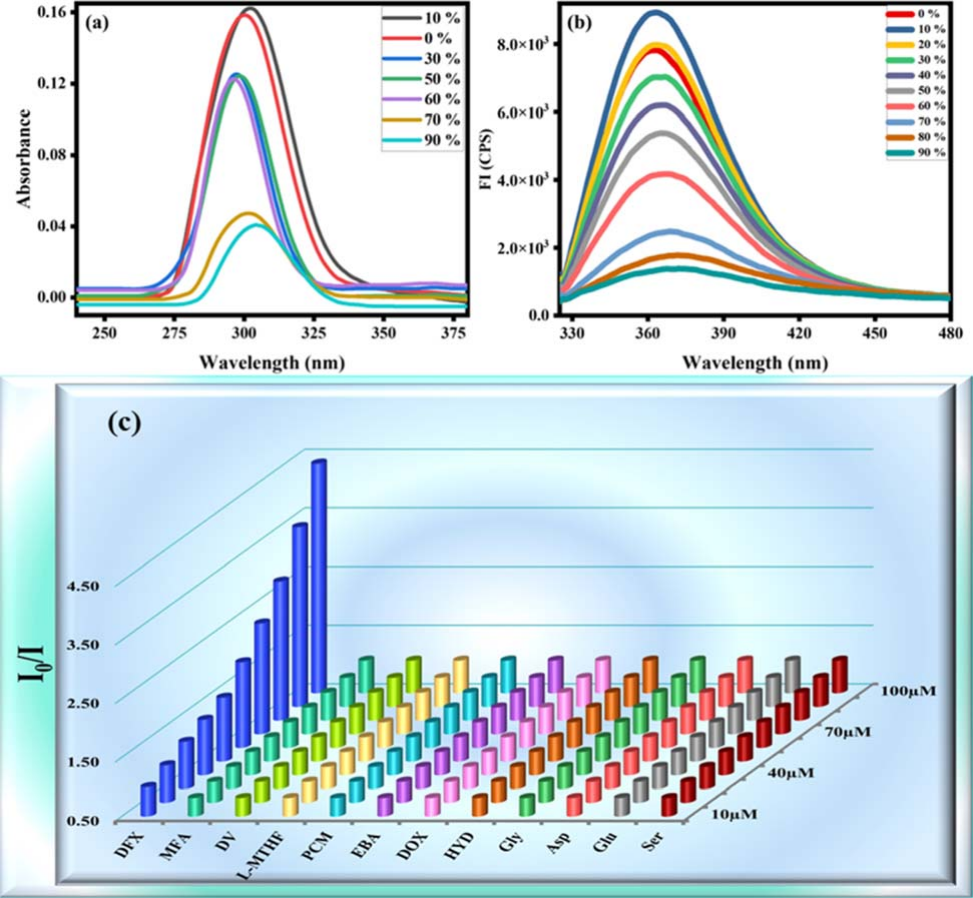
(a) Absorbance and (b) emission spectra of 3,4-dimethoxyaniline at different water fraction (c) 3D SV plot of probe MPT against various drugs.

Absorption and emission pattern of deferasirox were also monitored to ensure that the results of the sensor are intrinsic to the sensor instead of showing analyte's fluorogenic behaviour. Absorption and emission maxima of deferasirox were at 301 nm and 498 nm respectively (Fig. 1d). An extensive fluorescence experimentation of 3,4-dimethoxyaniline was also performed to understand how the fluorescent sensor and its one part are related. 3,4-Dimethoxyaniline showed sufficient fluorescence emission but its fluorescence gradually started diminishing upon aggregating with more than 20% water which makes it unsuitable to be used in biological system (Fig. 2a and b).

### Fluorescence sensing of DFX

**Fig. 3:**
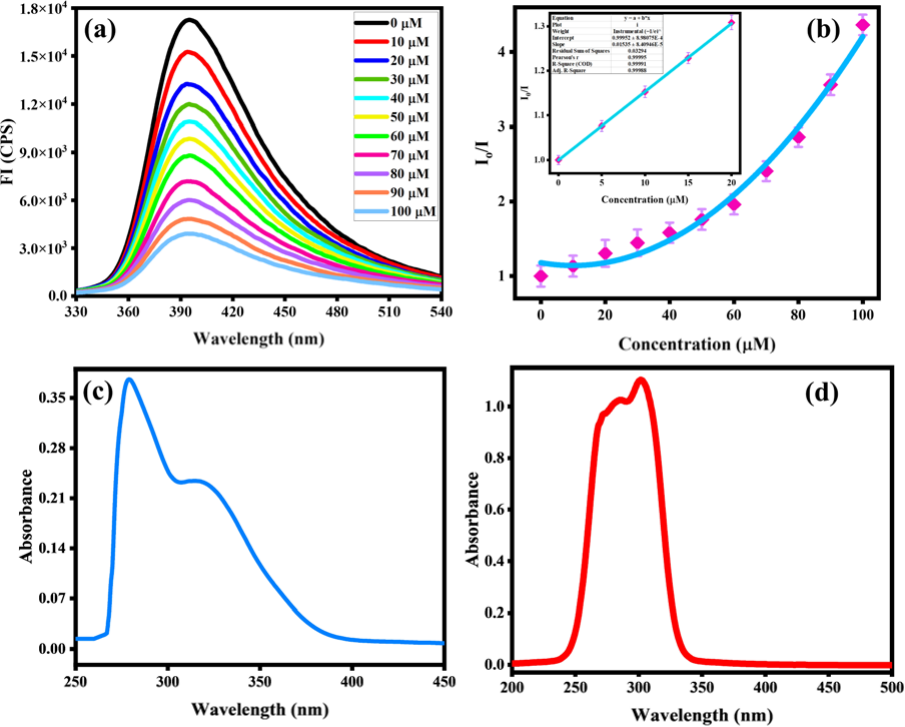
(a) Detection response of probe MPT against DFX (0–100 μM) at *λ*_max_ = 394 nm with 90% water fraction and (b) the SV plot of probe MPT against DFX at *λ*_ecx_ = 305 nm (c) absorption spectrum of MPT and (d) absorption spectrum of DFX.

The spectrofluorimetric analysis were undergone to study the detection potential of probe MPT toward deferasirox (DFX) in solvent system of water/THF, 9 : 1, v/v. This experiment was carried out by gradually adding (0–100 μM) of targeted analyte vis. deferasirox (DFX), mefenamic acid (MFA), drotaverine (DV), l-methyl folate (l-MTHF), paracetamol (PCM), ebastine (EBA), doxycycline (DOX), hydroxyurea (HYD), glycine (Gly) and aspartic acid (Asp). The emission intensity of probe MPT shows no significant change with the addition of all other analytes, which confirms the selectivity of deferasirox. The 3D Stern–Volmer was plotted which verified that the fluorescence emission intensity of MPT was selectively quenched by deferasirox (Fig. 2c). We observed the reduction in the emission intensity of MPT upon adding DFX. The probe MPT displayed strong emission intensity at 394 nm that quenched gradually upon increasing the concentration of DFX from 10 to 100 μM (Fig. 3a). The fluorescence titration experiments were undertaken to assess the binding efficiency of probe MPT with deferasirox (DFX).

The maximum quenching of fluorescence emission was observed at the 100 μM concentration of deferasirox (DFX) and the quenching efficiency was calculated to be 59%. Moreover, the SV constant was calculated through the Stern–Volmer equation that verified the selectivity of deferasirox (DFX) with probe MPT.*I*_o_/*I* = 1 + *K*_sv_[*Q*]where *I*_o_ is the emission intensity of pure MPT, *I* represent the emission intensity of probe MPT after the addition of deferasirox (DFX), *K*_sv_ denotes the Stern–Volmer constant, and [*Q*] symbolizes the amount of deferasirox (DFX). The relative intensities *I*_o_/*I* of probe MPT were plotted against increasing concentrations of deferasirox (DFX) to obtain the SV graph (Fig. 3b).

The SV graph showed the linear response of deferasirox (DFX) up to 20 μM while at high concentration from 20 μM to 100 μM SV plot showed the steep response of DFX with *K*_sv_ value of 3.4 × 10^4^ M^−1^ that indicate the strong interaction between MPT and DFX at higher concentration. This can be explained by the occurrence of a proliferation of molecules surrounding probe MPT hence significantly decreasing the emission intensity and showing positive deviation from SV. Quantum yield of MPT with varifying DFX concentration was calculated and observed to decrease at regular interval (Table S1a†). Similarly, the limit of detection (LOD) of 200 nM was calculated to estimate the sensitivity of probe MPT for deferasirox (DFX) by using the formula 3*σ*/*S*. The calculated LOD was significantly less than the already reported deferasirox (DFX) detection probe MPT listed in (Table S1b†). Further, the binding stoichiometry of probe MPT with deferasirox (DFX) was obtained from continuous variance calculation. For this purpose, equimolar solutions of probe MPT with deferasirox (DFX) were prepared. The binding stoichiometry of probe MPT and DFX was perceived through Job's plot which was plotted by increasing the mole fraction of deferasirox (DFX) and relative fluorescence emission. The 0.5 mole fraction of DFX turned out to show maximum fluorescence emission which is evident from Job's plot (Fig. S1†). The binding stoichiometry is 1 : 1 between probe MPT and deferasirox (DFX). ^1^H-NMR titration executed among probe MPT and analyte also assists the outcomes from Job's plot. Absorption spectra of MPT and DFX was also analyzed showing significant difference from *λ*_max_ of MPT that was observed at 394 nm (Fig. 3c and d).

**Fig. 4:**
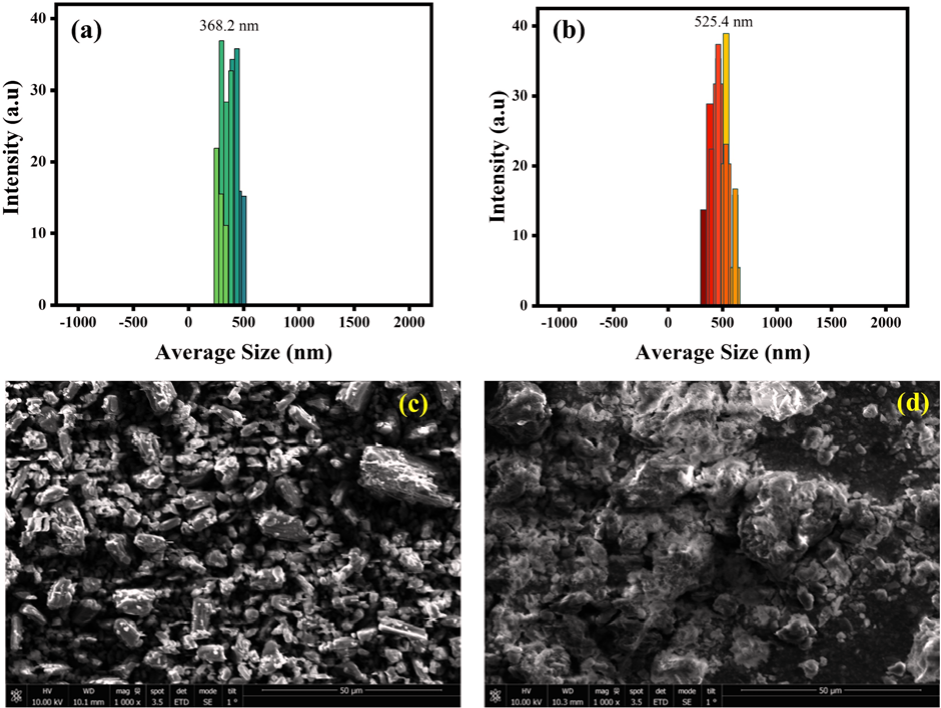
The DLS derived particle size distribution of (a) pure probe MPT and (b) MPT–DFX complex; SEM image of (c) probe MPT and (d) MPT–DFX complex.

Additionally, DLS analysis was also performed in support of fluorescence experiments to confirm the complex formation as the DLS technique is a powerful tool for determining particle size distribution in samples. Herein, the DLS experiment was executed to detect the change in the size of particles before and after the addition of DFX to the probe MPT in a solvent system of water/THF, 9 : 1. A considerable increase in particle size was identified as the average particle diameter of the probe MPT was 368.2 nm but after the addition of DFX the average particle diameter turned out to be 525.4 nm, consequently suggesting the formation of MPT–DFX complex (Fig. 4).

Moreover, the scanning electron microscopy (SEM) was also performed for probe MPT and MPT–DFX complex in order to monitor the morphological change before and after the formation of complex. In Fig. 4c, the independent microstructures of probe MPT are clearly visible while Fig. 4d shows the clusters of molecules with cloud-like structures after MPT–DFX complex formation.

### Interference studies

Interference analysis was performed through fluorescent emission studies to observe the selectivity of probe MPT for deferasirox (DFX). The 10 μM concentration of probe MPT was added to a solution containing DFX (50 μM) and some probably interfering species (Na^+^, K^+^, Li^+^, Mg^2+^, Co^2+^, Ni^2+^, Ca^2+^, Fe^2+^, Fe^3+^, Al^3+^, SO_4_^2−^, CH_3_COO^−^, CN^−^, F^−^, Cl^−^) (100 equiv.) (Fig. S2†). The selective quenching response of probe MPT towards DFX remains ineffective despite the existence of high concentrations of interfering species (100 equiv.). This unique selectivity of probe MPT for DFX makes it an appropriate contestant to be utilized in real-time applications.

Photostability is among some important factors that affect the probe MPT sensing capabilities that define real-time applicability. The probe MPT was treated with high-energy radiation to observe its photostability. The emission intensity of probe MPT (10 μM) toward DFX was investigated after being irradiated with high energy radiation (Fig. S3†) The quenching efficiency of probe MPT with DFX is insensitive to the high energy excited radiation that ensures excellent photostability. The emission response of probe MPT is also unaffected by varying pH and temperature. Therefore, emission spectra of probe MPT with DFX were obtained at different pH (Fig. S4a†) and a temperature range of 20 °C to 60 °C (Fig. S4b†). No considerable change was detected in the fluorescence emission of MPT with DFX; hence it can confirm the sensing of DFX at a wide range of pH and temperature. Similarly, the effect of time was also revealed by obtaining the emission spectrum of probe MPT at different concentrations of DFX and different intervals of time from 8 to 48 hours (Fig. S5a†) and 10 to 60 s (Fig. S5b†). The emission spectra indicate that the probe MPT shows the same quenching toward the DFX at different intervals of time. The results show that the quenching response of probe MPT towards DFX is not dependent on time.

### Detection mechanism for deferasirox (DFX)

**Fig. 5:**
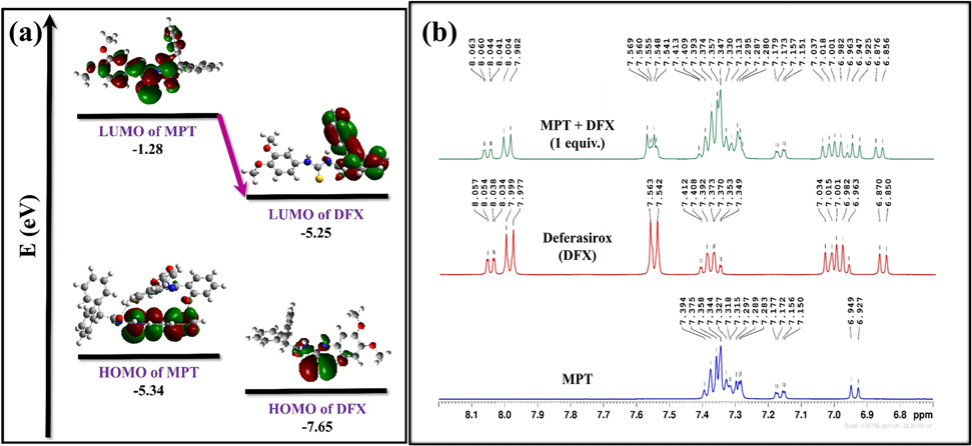
(a) FMO-based explanation of charge transfer and (b) ^1^H-NMR titration of probe MPT, DFX, and MPT + DFX in CDCl_3_.

The ^1^H-NMR titration experiment was used to examine the possible sensing mechanism by evaluating the type of interaction between probe MPT and DFX. First of all, the ^1^H NMR spectrum of probe MPT and DFX were taken separately. After that, the ^1^H-NMR spectrum of the MPT–DFX complex was obtained in 1 : 1 concentration and spectra were analyzed to understand the strength of interaction between probe MPT and DFX. The ^1^H-NMR titration results showed that there was no substantial change in chemical shifts or multiplicity before and after the formation of the MPT–DFX complex. A doublet of probe MPT from 6.927 and 6.949 ppm shifted upfield to 6.925 and 6.947 ppm. Similarly, another doublet of doublet between 7.150 and 7.177 shifted to 7.151 and 7.179 ppm clarifying the NCI between the probe MPT and DFX (Fig. 5b).

Optical characteristics of the MPT–DFX complex were revealed by using UV-vis spectroscopy. The probe MPT showed maximum absorption *λ*_abs_ at 300 nm in UV-vis spectrum. Then after the addition of DFX in probe MPT, UV-vis spectrum showed only a minor decrease in intensity at *λ*_abs_ with no bathochromic or hypso-chromic shift (Fig. S6†). Correspondingly, in the fluorescence experiment, with the accumulation of DFX in probe MPT, no bathochromic shift or hypso-chromic shift in *λ*_max_ (at 394 nm) along with no emergence of any new peak. On the addition of DFX in the probe MPT quenching was detected in the fluorescence emission intensity (Fig. S7†). Both the UV-vis and fluorescence spectra results also suggest that the primary interaction between MPT and DFX was non-covalent.

**Fig. 6:**
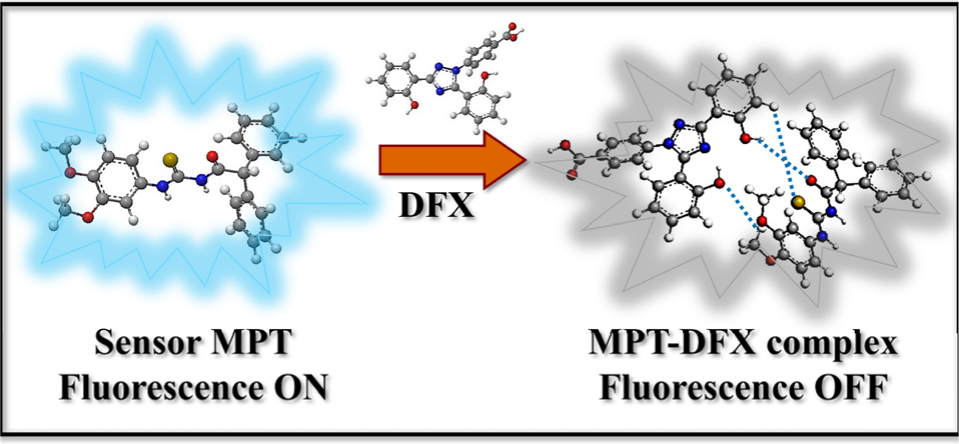
Diagram illustrating the sensing mechanism used to detect DFX.

To get further insight into the origin of sensing DFX through probe MPT, plausible sensing mechanisms responsible for the detection of DFX were investigated. The emission intensity of probe MPT was considerably quenched upon successively adding DFX to it as illustrated through the fluorescence titration experiments. Quenching in emission intensity of probe MPT could be due to the transfer of photoexcited electron transfer from donor to acceptor or Förster resonance energy transfer (FRET) between two interacting moieties. FRET mechanism is predicted by the overlap of absorption and emission spectra of the analyte and probe respectively.^53^ The absorption spectra of the analyte are in the range of 250 nm to 350 nm while the probe MPT shows emission at *λ*_max_ at 394 nm (Fig. S8†). A very negligible and insignificant overlap of the absorption spectra of DFX and the emission spectrum of MPT excluded the prospect of the existence of FRET mechanism. The possible mechanism is photo-induced electron transfer (PET) which was further supported through NBO analysis by using the DFT methodology. NBO revealed that the −0.02687e^−^ charge was transferred from probe MPT to DFX. Further, the energy gap between HOMO–LUMO orbitals of probe MPT and complex was calculated through FMO analysis. The LUMO of probe MPT was situated at −1.28 eV while the LUMO of DFX resided at −1.60 eV. As the LUMO of probe MPT was found at higher energy as compared to the LUMO of DFX that indicates that charge transfer occurs from probe MPT to DFX (Fig. 5a). To further elucidate the types of interactions between probe MPT and DFX, NCI analysis was carried out which confirmed the van der Waals interactions between MPT and DFX. The weak van der Waals forces between the probe MPT and DFX were basically comprised of π–π stacking of phenyl rings of the probe MPT and DFX are responsible for these weak van der Waals interactions (Fig. 6). These experimental and theoretical studies confirm the interaction through charge transfer between MPT and DFX which can be from the phenolic group of DFX to probe MPT.

### Theoretical studies

#### Geometric optimization

The non-covalent interactions and the most thermodynamically stable site of interaction between the probe MPT and DFX were evaluated by calculating the interaction energies (*E*_int_) at various feasible interfaces. The site of interaction of MPT–DFX that has greater interaction energy is more thermodynamically stable and conversely. The computed interaction energies at three different sites I, II and III (Fig. S9†) were calculated as −19.03, −29.05, and −25.27 kcal mol^−1^ correspondingly. The highest *E*_int_ value was calculated to be −29.05 kcal mol^−1^. Therefore, site II was found to be the optimized interaction site in the MPT–DFX complex.

#### Electronic properties

**Fig. 7:**
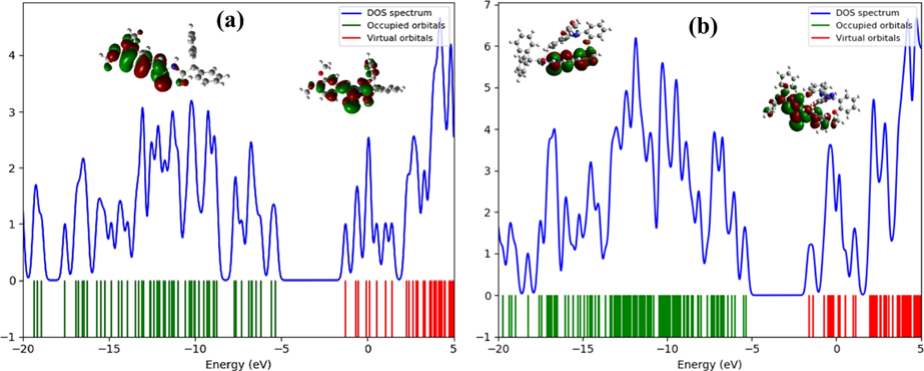
(a) Representation of density of states (DOS) spectra of probe MPT and (b) MPT–DFX complex.

The efficiency of the probe towards detecting the analyte is explained by the electronic properties of interacting compounds. HOMO–LUMO energy gap reduction depicts good sensitivity while its expansion depicts resistivity. HOMO of the probe MPT is usually electron rich and donates the electronic charge to LUMO of analyte which is electron deficient resulting in HOMO–LUMO energy gap reduction. Electronic properties of probe MPT, before and after the formation of MPT–DFX complex, were investigated and a prominent change in electronic properties was observed. The probe MPT exhibited HOMO–LUMO energy gap of 4.06 eV while the HOMO level was situated at −5.34 eV and the LUMO level at −1.28 eV. The HOMO–LUMO energy gap turned out to be 3.70 eV after MPT–DFX complex formation while the HOMO and LUMO levels were shifted to −5.33 and −1.63 eV respectively. Moreover, the DOS analysis confirmed the good sensitivity of the MPT against DFX as the HOMO–LUMO energy gap was reduced and new virtual energy levels appeared in DOS spectra (Fig. 7).

#### Exploration of non-covalent interactions (NCI)

**Fig. 8:**
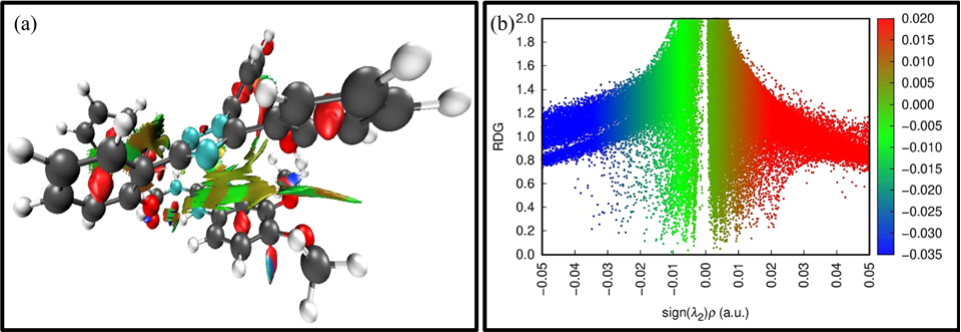
(a) Illustration of 3D and (b) 2D NCI plots of MPT–DFX complex.

The NCI analyses with 3D isosurface and 2D-RDG were performed to investigate the type of interaction between the probe MPT and DFX. Colour contours explain the type of interaction in 3D isosurface as green patches represent weak forces such as van der Waals forces, red areas depict steric repulsions and blue colour is representative of strong interactive forces, for instance, dipole–dipole interactions and H-bonding. The definite green colour at the interaction site in 3D isosurface of the MPT–DFX complex depicts the π–π forces of attraction present in it. Furthermore, strong H-bonding is evident from the blue patches observed between acidic hydrogen of phenol functionality in DFX and carbonyl oxygen as well as oxygen of methoxy group of probe MPT. Moreover, in the 2D NCI plot, the nature of collaboration among the probe MPT and analyte DFX was also represented by values of sign *ρ* (*λ*2) (*x*-axis) against which RDG (*y*-axis) was plotted. The repulsive interactions are shown when values of the sign *ρ* (*λ*2) are greater than zero while lower than zero values of *ρ* (*λ*2) show strong attractive interactions between the MPT–DFX. The presence of green spots indicated weak non-covalent interactions such as π–π stacking. Also, the bluish-green to blue spikes in the −0.015 to 0.035 region correspond to the weaker to stronger H-bonding (Fig. 8).

#### NBO and EDD analysis

EDD provides visual assistance in the characterization of non-covalent intermolecular forces and NBO analyses helped estimate the transfer of charge between the probe MPT and the analyte DFX, to recognize the sensing mechanism of the probe against the analyte and to assess the sensitivity and characteristic properties of the probe MPT. Charge transfer is visually displayed by EDD while the magnitude of charge transfer is explained *via* NBO. The MPT–DFX complex showed indigo patches in the 3D isosurface which depicted the accumulation of charge densities and purple patches revealed the depletion of charge density (Fig. S10†). Indigo tones in the EDD graph showed the intensification of electronic density in those regions where the atoms of the probe and the analyte interacted which confirmed the consequences of FMO. Furthermore, the magnitude of the NBO charge for MPT after interaction with DFX was −0.02687e^−^.

#### QTAIM analysis

Bader *et al.* first proposed the QTAIM analysis to analyze the type of non-covalent interaction and bonds between interacting moieties.^52^ In QTAIM, the interaction type is evaluated through bond critical points (BCPs) *via* some topological parameters including Laplacian of the charge density (∇^2^*ρ*), total energy density (*H*(*r*)), total electron density (*ρ*(*r*)), potential energy density (*V*(*r*)), kinetic energy density (*G*(*r*)) and individual bond's interaction energy (*E*_int_). Less than zero values of total energy density and Laplacian of the charge density show covalent interaction while positive non-zero values indicate non-covalent interaction. Additionally, other parameters such as the ratio of *V*(*r*) to *G*(*r*) also assist in analyzing the types of interaction. If its value is less than 1, it indicates the non-covalent interactions while the value greater than 2 corresponds to the covalent interaction between the probe and the analyte. The eight BCPs were obtained for the MPT–DFX complex (Fig. 8a). The value range of *ρ*(*r*) and ∇2*ρ*(*r*) turned out to be 0.0054–0.0275 and 0.0177–0.0937 a.u. respectively. The strong H-bonding between H of MPT and O of the DFX was demonstrated by the large values of *ρ* (0.0349 a.u.) and ∇2*ρ* (0.1081 a.u.). The −*V*(*r*)/*G*(*r*) values showed the strength of interaction as they were greater than 1 but smaller than 2 which denoted the partially covalent H-bonds. Likewise, a very slight covalent H-bonding between S⋯H, H⋯O and O⋯H atoms of the MPT–DFX complex is recognized from the negative values of *H*(*r*). Moreover, the other values of topological parameters obtained from QTAIM analyses include *G*(*r*), *V*(*r*), −*V*(*r*)/*G*(*r*), and *H*(*r*) which assisted in identifying the weak electrostatic forces between the probe and the analyte are shown in Table S3.†

#### SAPT0 analysis

**Fig. 9:**
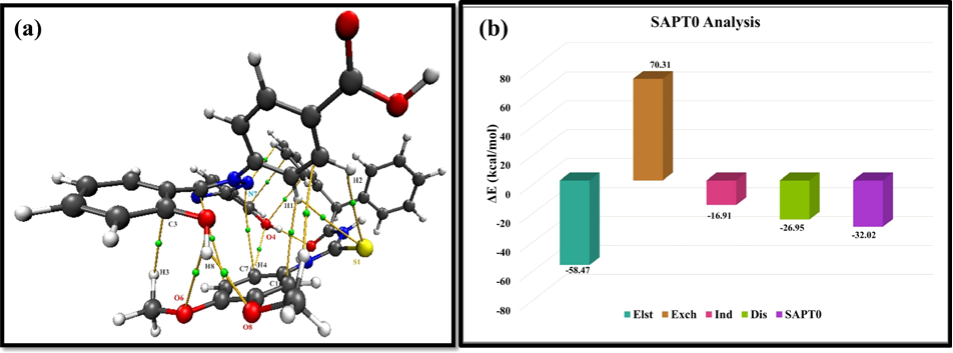
(a) Bond critical points between MPT–DFX shown by QTAIM results and (b) representation of SAPT0 interaction energies.

Quantification of four different components (Δ*E*_elst_, Δ*E*_exch_, Δ*E*_ind_, and Δ*E*_disp_) of interaction energy was carried out with SAPT0 analysis. Δ*E*_elst_ elaborates the interaction between MPT and DFX, induction (Δ*E*_ind_) calculates the extent of interaction in HOMO–LUMO orbitals of MPT and DFX, whereas dispersion (Δ*E*_disp_) explains the weak van der Waals's interactions. The value of Δ*E*_elst_, Δ*E*_ind_, and Δ*E*_disp_ are negative that indicates that interaction between the MPT and DFX is attractive. Positive value of Δ*E*_exch_ could be due to repulsive forces generated as a result of interaction between filled orbitals of MPT and DFX. Δ*E*_SAPT0_ for MPT and DFX was found to be −32.02 kcal mol^−1^ (Fig. 9b).

## Practical applications

### Rapid visual detection of DFX under UV

Visual detection experiments were undergone with the intent to confirm the selectivity of the probe MPT against DFX. Bright fluorescence emission of the probe MPT (10 μM) was observed under the UV 365 nm. Conversely, upon adding DFX (100 μM) into the probe solution emission was considerably quenched (Fig. S11†). However, no noticeable changes occurred upon the interaction with other drugs and amino acids, hence confirming the selectivity of the probe MPT towards DFX.

### Probe-coated test strips

**Fig. 10:**
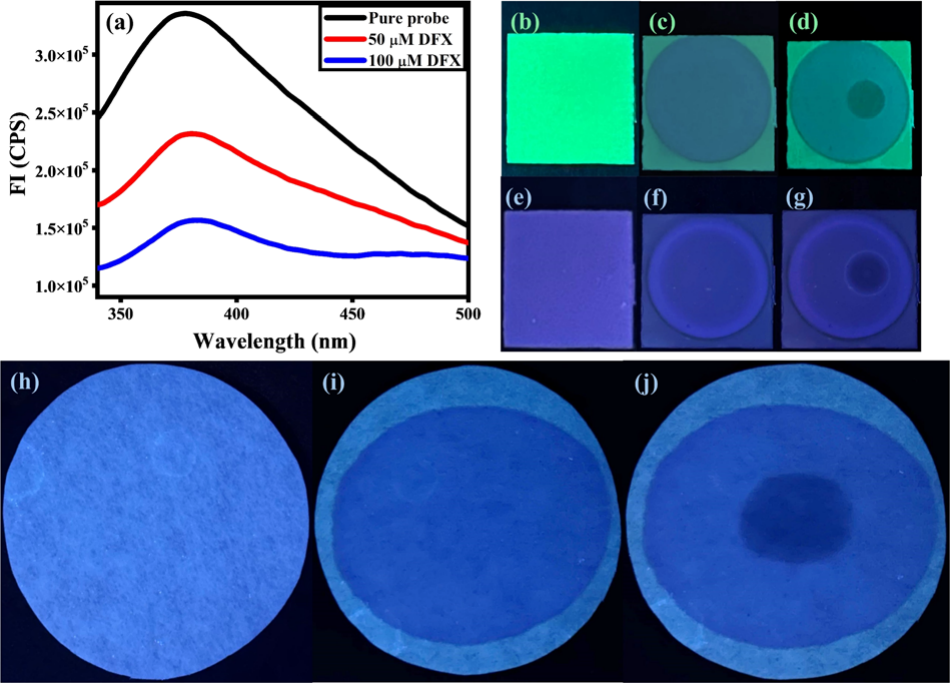
(a) Solid-state fluorescence emission, (b) plain TLC strip under UV 254 nm, (c) MPT-coated TLC strip under UV 254 nm, (d) DFX caused fluorescence quenching of MPT shown under UV 254 nm, (e) plain TLC strip under UV 365 nm, (f) MPT-coated TLC strip under UV 365 nm, (g) DFX caused fluorescence quenching of MPT shown under UV 365 nm, (h) plain Whatman filter paper under UV 365 nm, (i) MPT-coated Whatman filter paper under UV 365 nm and (j) DFX caused fluorescence quenching of MPT on Whatman filter paper shown under UV 365 nm.

The probe-coated low-cost test strips were designed so that the sensing ability of MPT against DFX could be explored. The solution of the probe MPT (50 μM) was poured onto the TLC plates and the TLC plates were dried in drying oven at 50 °C. The probe coated TLC strips showed fluorescence emission in UV (365 nm). As one drop of the analyte DFX was added to the TLC plate, a dark spot appeared showing the quenching of emission. Likewise, paper strips were also fabricated for further investigation of the sensing potential of MPT against DFX. In this aspect, a solution of 100 mM water/THF (9 : 1, v/v) was poured onto the Whatman filter paper strips and was dried at 50 °C in the drying oven for 10 minutes. The probe-coated paper strip showed emission in UV (365 nm) and emission change was observed upon the addition of DFX on it with the help of a micropipette (Fig. 10a). Visible difference in emission upon the addition of 50 μM DFX onto fabricated paper strip and TLC strips verified its potential to be used for on-site, rapid and low-cost sensing of DFX (Fig. 10b–j).

### Detection of DFX in plasma

Fluorescence spectroscopic experiments were undertaken to assess the practicality of the probe MPT for detecting DFX in human plasma. In that respect, plasma was spiked with concentrations of DFX (20–100 μM). The emission intensity of the 40 μM solution of MPT was quenched noticeably upon adding the known concentration of DFX containing plasma samples in it (Fig. S12a†). The recoveries of DFX spiked samples turned out to be with RSD (relative standard deviation) ranging from 1.18–1.32% are shown in Table S3.† The practicality of probe MPT for sensing DFX in plasma is proved valid by the results.

### Detection of DFX in real water samples

Fluorescence sensing was also carried out on real water samples spiked with known concentrations (20–120 μM) of DFX. Upon successive addition of spiked water samples into probe MPT, quenching was observed (Fig. S12b†). Experiments were carried out thrice and RSD (relative standard deviation) was calculated as 1.17–1.29%. The results discussed in Table S4† confirm that probe MPT can be used for detection in real water samples.

### Detection of DFX in artificial urine samples

Artificial urine was prepared in the laboratory by mixing urea (250 mmol), uric acid (1.48 mmol), creatinine (7.79 mmol), citrate (2.45 mmol), sodium (92.62 mmol), potassium (31.33 mmol), ammonium (23.66 mmol), calcium (1.66 mmol), magnesium (4.38 mmol), chloride (88.00 mmol), oxalate (0.277 mmol), sulphate (18 mmol) and phosphate (23.33 mmol) in 1.5 litre double distilled water at 37 °C with continuous stirring. The artificially prepared urine was then spiked with different concentrations of DFX (2–20 μM) and monitored through fluorescence which quenched upon increasing concentrations of DFX as shown in Fig. S13.† Triplicate experiments were carried out with a relative standard deviation calculated as 0.28–0.52% (Table S5).†

## Conclusions

In conclusion, the fluorescence probe MPT was utilized for efficient sensing of the deferasirox in real samples. Probe MPT was characterized through ^1^H and ^13^C NMR spectroscopy. The effective non-covalent interaction between probe MPT and DFX was confirmed through ^1^H-NMR, UV-vis, and fluorescence spectroscopy. The excellent selectivity of the probe MPT toward DFX was demonstrated through PET-mediated unique quenching response in fluorescence emission intensity of probe MPT in the presence of various equivalents of a wide range of interfering species under different conditions. Further, aggregate formation and the complexation of probe MPT with DFX was confirmed by DLS analysis and SEM technique. The DFT calculations supported the presence of weak intermolecular forces and thermodynamic stability of the MPT–DFX complex. NCI results revealed the existence of weak to strong intermolecular interactions. FMO and DOS analysis confirmed the sensitivity of probe MPT for the sensing of DFX. EDD and NBO analyses showed the transfer of charge from probe MPT to DFX while QTAIM and SAPT0 analysis confirmed all possible interactions between individual atoms of MPT–DFX complex. The real-time applications of fluorescence probe MPT for DFX detection in plasma and real water samples demonstrated the practical utility of MPT fluorescent probe.

## Data availability

The data supporting this article have been included as part of the ESI.†

## Author contributions

Kainat Khurshid: methodology, investigation, validation, writing – original draft. Sohail Anjum Shahzad: supervision, conceptualization, methodology, visualization, validation, investigation, project administration, funding acquisition, writing – review & editing. Mohammed A. Assiri: visualization, methodology, software, formal analysis. Alam Shabbir: methodology, investigation, formal analysis, writing – original draft. Tayyeba Javid: methodology, investigation, formal analysis. Hasher Irshad: investigation, conceptualization, methodology, validation.

## Conflicts of interest

The authors declare that they have no known competing financial interests or personal relationships that could have appeared to influence the work reported in this paper.

## Supplementary Material

RA-014-D4RA03548H-s001
