# Supplementary material for: Highly sensitive AIEE active fluorescent probe for detection of deferasirox: extensive experimental and theoretical studies
Source: RSC Adv. 2024 Jul 8;14(30):21682–91. doi: 10.1039/d4ra03548h (PMC11228937; doi:10.1039/d4ra03548h)

Electronic Supplementary Material (ESI) for RSC Advances.
This journal is © The Royal Society of Chemistry 2024

Supporting Information

Highly Sensitive AIEE Active Fluorescent Probe for Detection of Deferasirox: Extensive Experimental and Theoretical Studies

Kainat Khurshid[a], Sohail Anjum Shahzad[a,*], Mohammed A. Assiri[b,c], Alam Shabbir[a], Tayyeba Javid[a], Hasher Irshad[a]

[a] *Department of Chemistry, COMSATS University Islamabad, Abbottabad Campus, University Road, Abbottabad 22060, Pakistan*

[b] *Department of Chemistry, Faculty of Science, King Khalid University, P.O. Box 9004, Abha 61413, Saudi Arabia*

[c] *Research Center for Advanced Materials Science (RCAMS), King Khalid University, Abha 61514, P. O. Box 9004, Saudi Arabia*

* **Corresponding author:** Sohail Anjum Shahzad, Department of Chemistry, COMSATS University Islamabad, Abbottabad Campus, University Road, Abbottabad 22060, Pakistan.

E-mail addresses: sashahzad@cuiatd.edu.pk & sohail_chem@yahoo.com (S. A. Shahzad)

Table of Contents

SI-1. Instruments and reagents 3

Fig. S 1. Jobs plot of sensor **MPT**-DFX complex in H_2O:THF (9:1, v/v) 3

Fig. S 2. The emission response of sensor **MPT** towards DFX before and after adding some common interferences 4

Fig. S 3. Photostability test of probe **MPT** 4

Fig. S 4. (a) Effect of pH and (b) Effect of temperature on the relative fluorescence quenching of sensor **MPT** towards DFX 5

Fig. S 5. (a) Relative fluorescence quenching of **MPT** for DFX in the time interval of 8 – 48 hours and (b) Relative fluorescence quenching of **MPT** for DFX in the time interval of 10 – 60 sec. 5

Fig. S 6. UV-*Vis.* absorbance of **MPT**-DFX 6

Fig. S 7. UV-*Vis* absorbance and fluorescence emission of sensor **MPT** in THF solution (10 μM) 6

Fig. S 8. The absorbance of DFX and emission spectra of **MPT** 7

Fig. S 9. Optimized geometries of sensor **MPT** (a) Site I **MPT**-DFX (b) Site II **MPT**-DFX (c) Site III **MPT**-DFX 7

Figure S 10. Electron density difference image of **MPT**-DFX Complex 8

Fig. S 11. Bright fluorescence of **MPT** (a) before adding DFX and, (b) after adding DFX 8

Fig. S 12. (a) Fluorescence relative emission of sensor **MPT** in plasma and (b) Real water sample. 9

Fig. S 12 Fluorescence quenching observed in spiked artificial urine samples.. **Error! Bookmark not defined.**

Table. S 1. (a) The quantum yield of MPT in different water fractions and with DFX **(b)** Comparison of sensor **MPT** with already reported DFX sensors 10

Table. S 2. QTAIM analysis of **MPT**-DFX 11

Table. S 3. Spike and recovery experiment for detection of DFX in plasma 11

Table. S 4. Spike and recovery values for DFX detection in real water samples 12

Table S 5. Spike and recovery values for the detection of DFX in artificial urine samples 13

References: 14

SI-2. NMR spectra of the synthesized compound .... **Error! Bookmark not defined.**

SI-1. Instruments and reagents

The sensor **MPT** was characterized by performing ^1H NMR at 400 MHz, ^{13}C NMR and DEPT-135 at 100 MHz with the help of Bruker Avance III NMR spectrometer employing $CDCl_3$ as solvent and the reference compound was TMS (tetramethylsilane). NMR titration experiments were carried out in $CDCl_3$. The spectrofluorometer (FluoroMax-Plus-P-C, Horiba Jobin Yvon Technology, USA) was used to record the fluorescence emission spectra. The fluorescence studies were performed on solutions of sensor **MPT** in HPLC grade THF in order to detect the anticipated analyte. All chemicals and reagents, purchased from Daejung Chemicals & Metals (Korea), Oakwood Chemicals (USA), Sigma Aldrich (USA), and Alfa Aesar (UK), were employed directly without any further purification. Reagents and chemicals included diphenylacetic acid, potassium thiocyanate, 3,4 dimethoxyaniline, THF, dichloromethane, methanol, sodium bicarbonate, and distilled water. The plasma (P9523, MDL number: MFCD00131920) was purchased from Sigma Aldrich (USA).

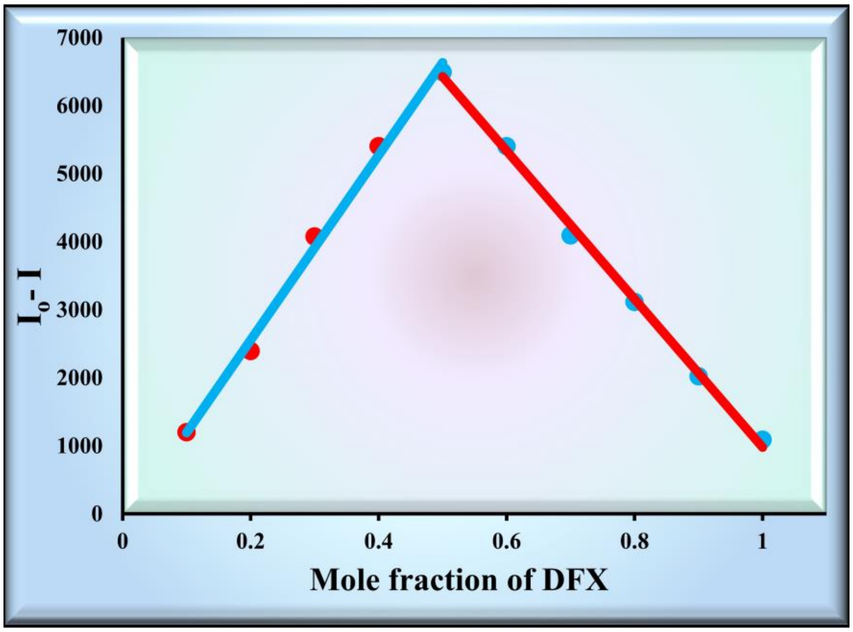

Fig. S 1. Jobs plot of sensor **MPT**-DFX complex in H_2O:THF (9:1, v/v)

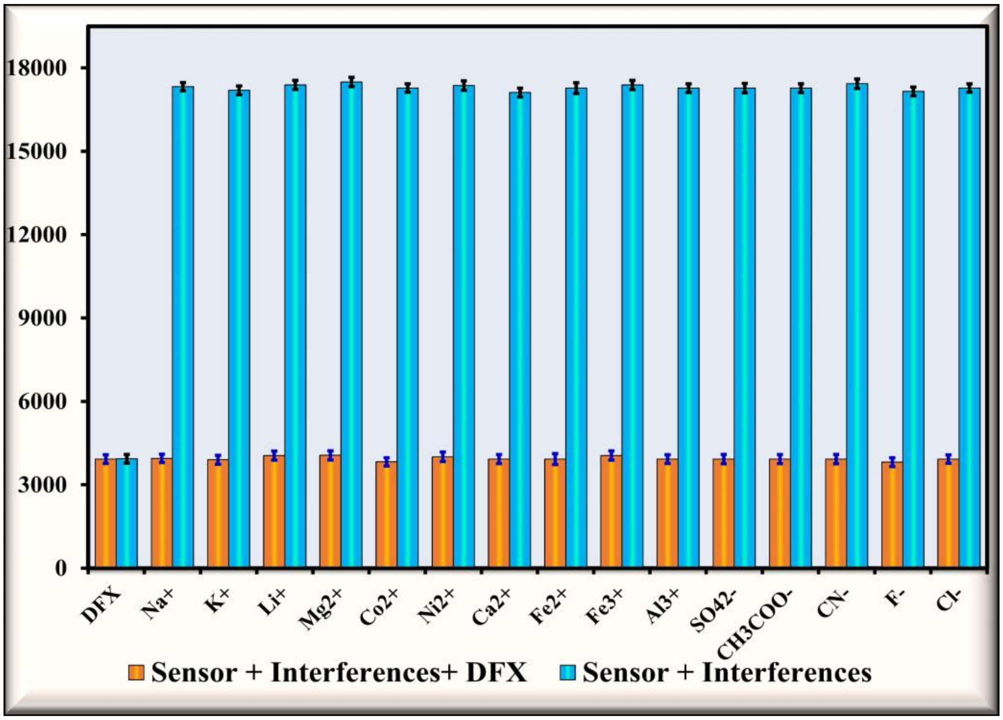

Fig. S 2. The emission response of sensor **MPT** towards DFX before and after adding some common interferences

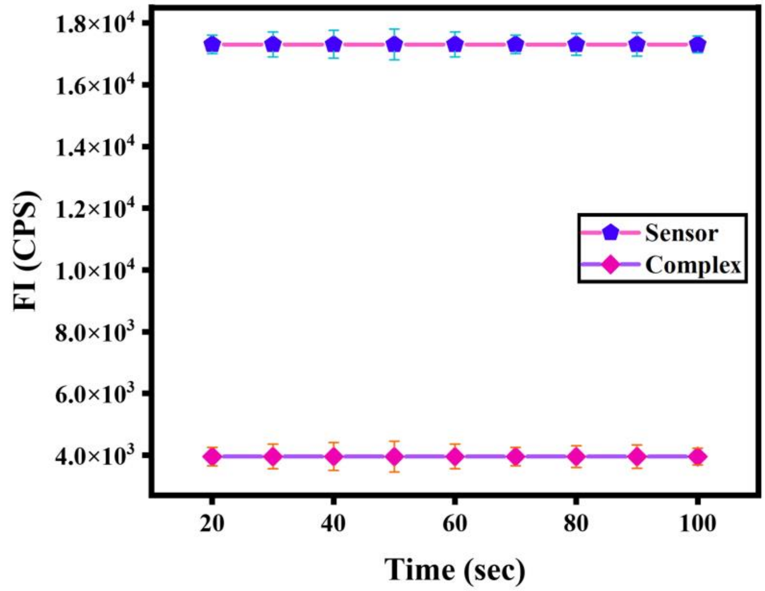

Fig. S 3. Photostability test of probe **MPT**

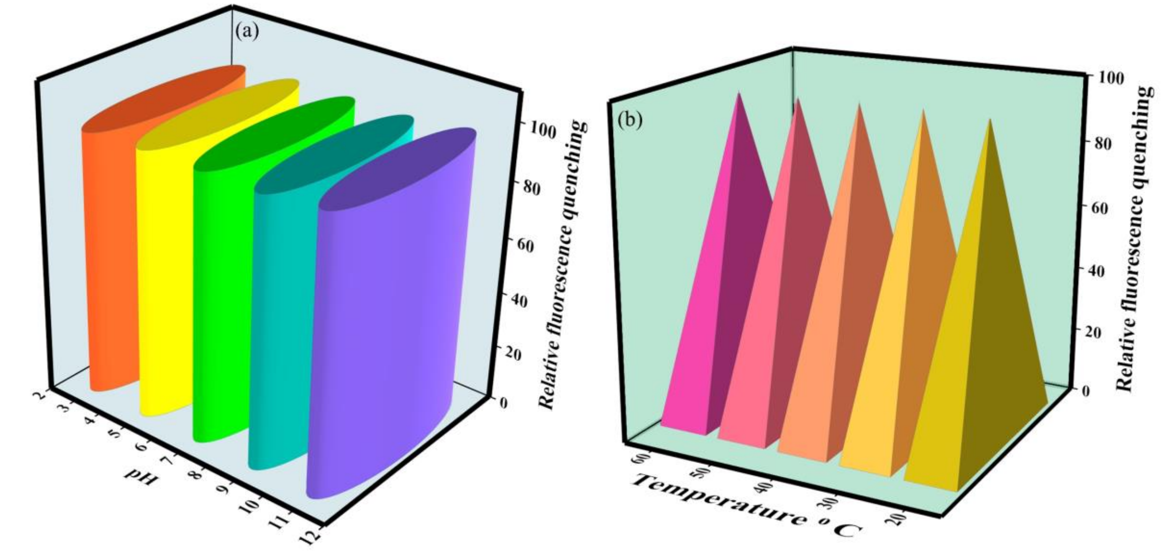

Fig. S 4. (a) Effect of pH and (b) Effect of temperature on the relative fluorescence quenching of sensor **MPT** towards DFX

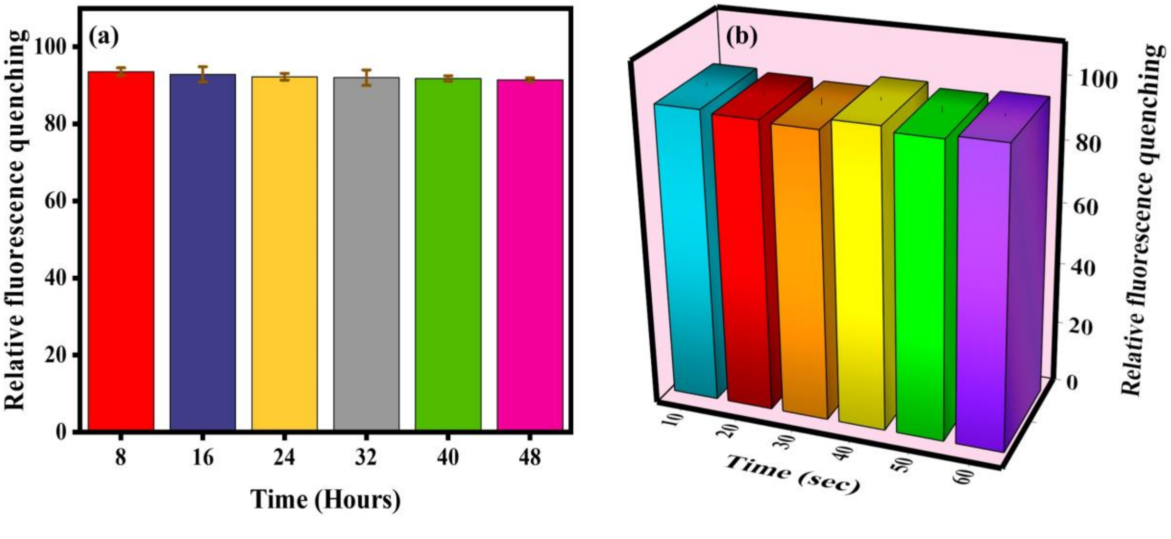

Fig. S 5. (a) Relative fluorescence quenching of **MPT** for DFX in the time interval of 8 – 48 hours and (b) Relative fluorescence quenching of **MPT** for DFX in the time interval of 10 – 60 sec.

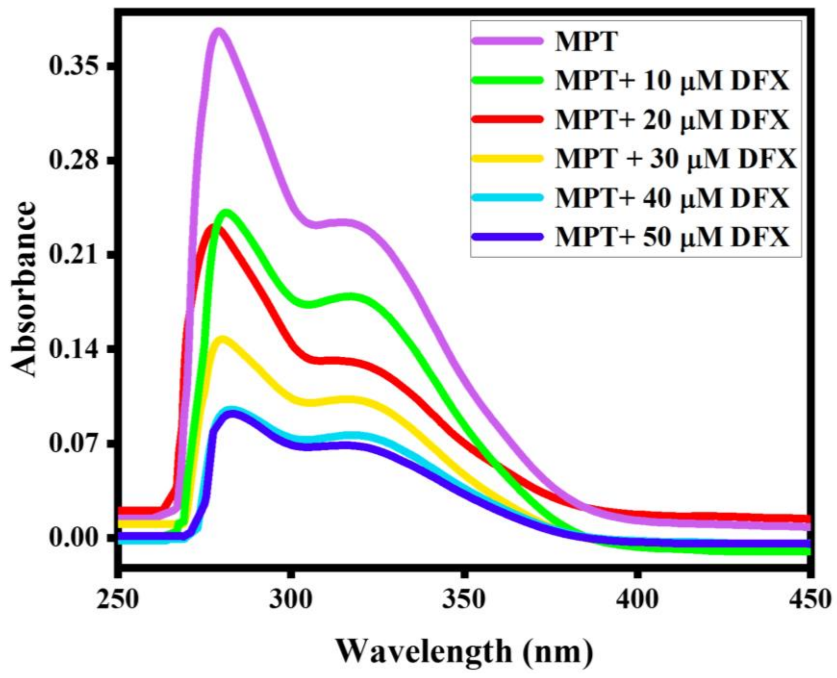

Fig. S 6. UV-*Vis.* absorbance of **MPT**-DFX

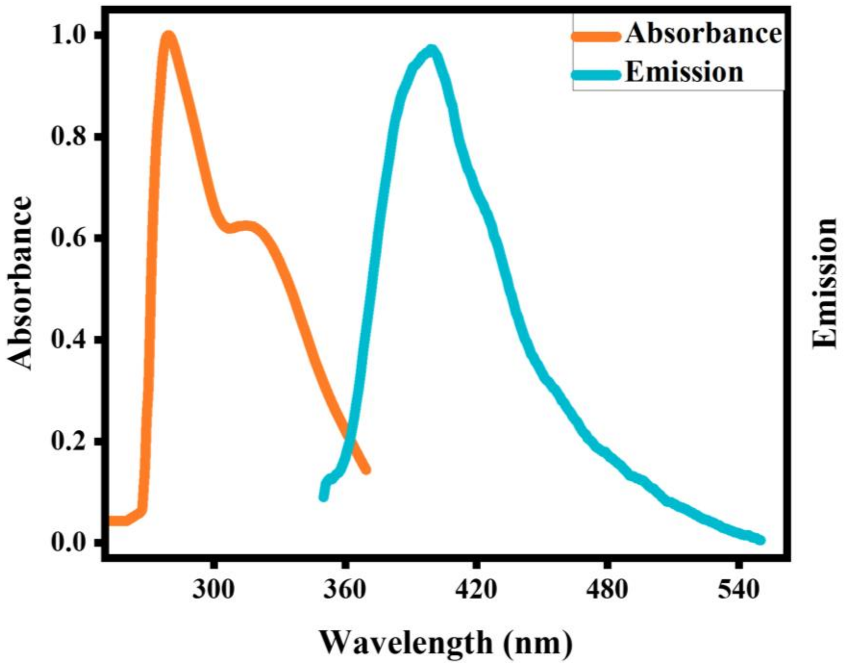

Fig. S 7. UV-*Vis* absorbance and fluorescence emission of sensor **MPT** in THF solution (10 µM)

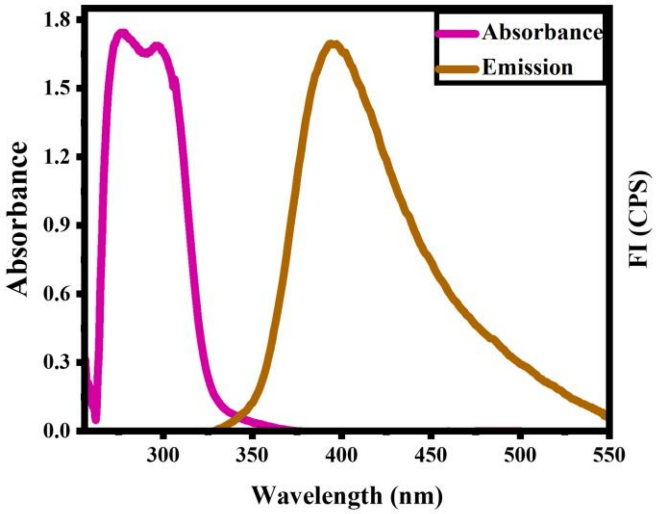

Fig. S 8. The absorbance of DFX and emission spectra of **MPT**

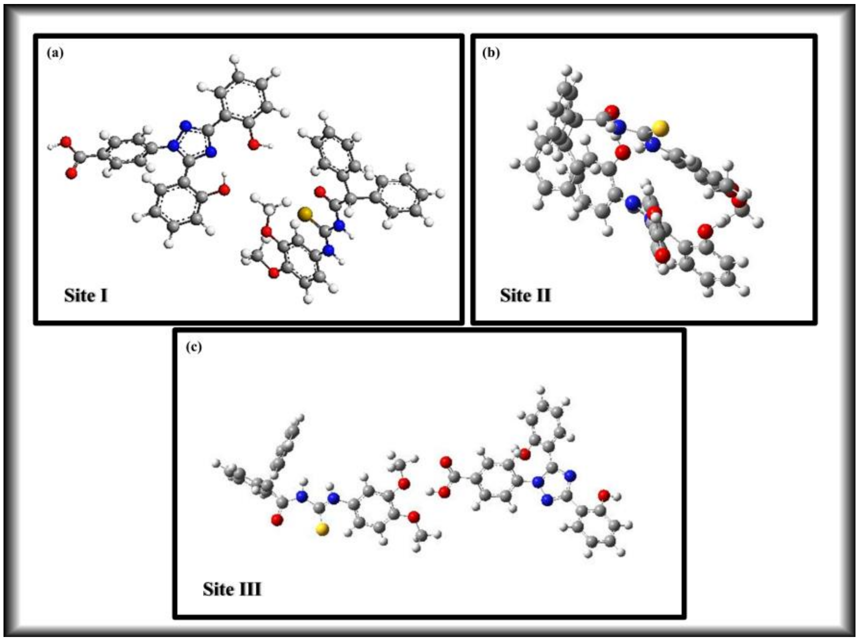

Fig. S 9. Optimized geometries of sensor **MPT** (a) Site I **MPT**-DFX (b) Site II **MPT**-DFX (c) Site III **MPT**-DFX

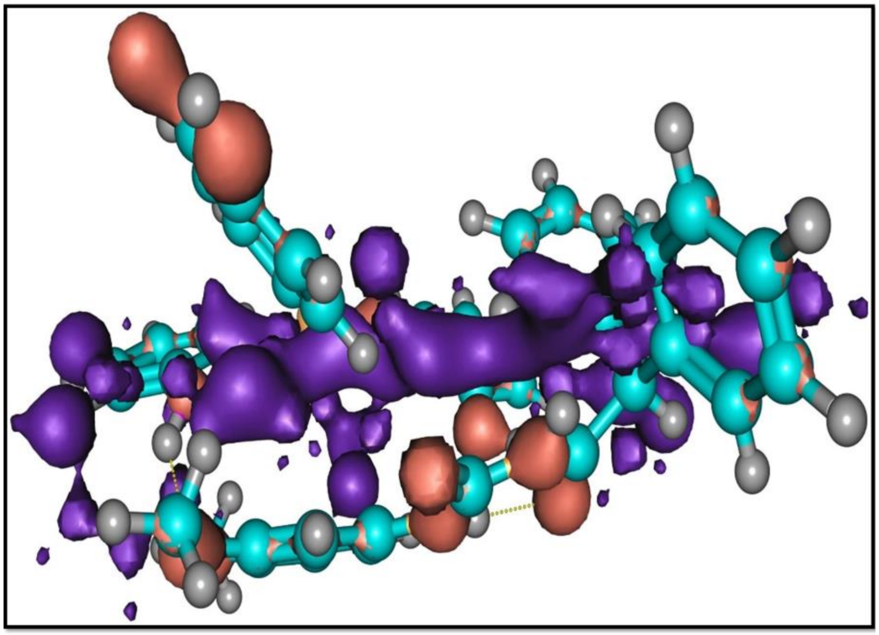

Figure S 10. Electron density difference image of **MPT**-DFX Complex

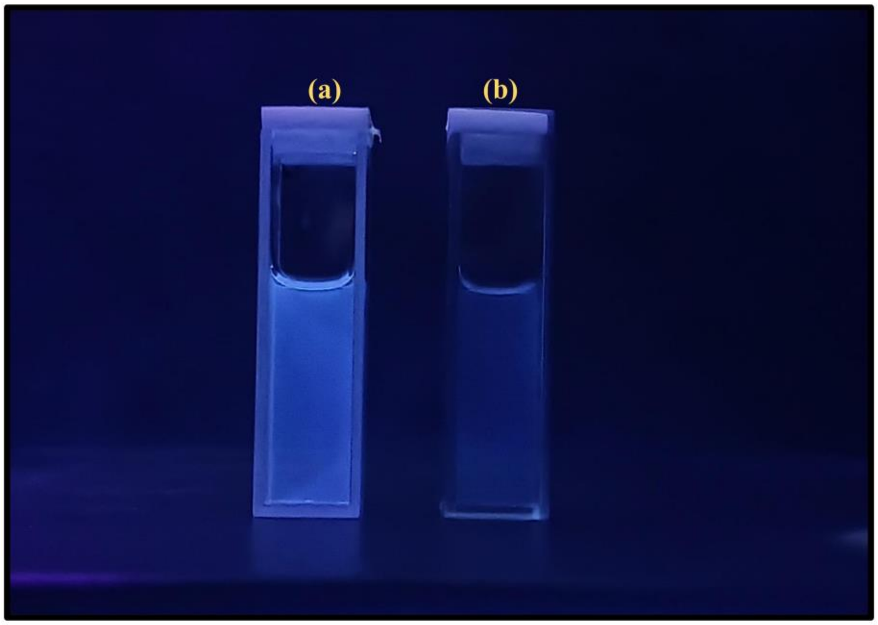

Fig. S 11. Bright fluorescence of **MPT** (a) before adding DFX and, (b) after adding DFX

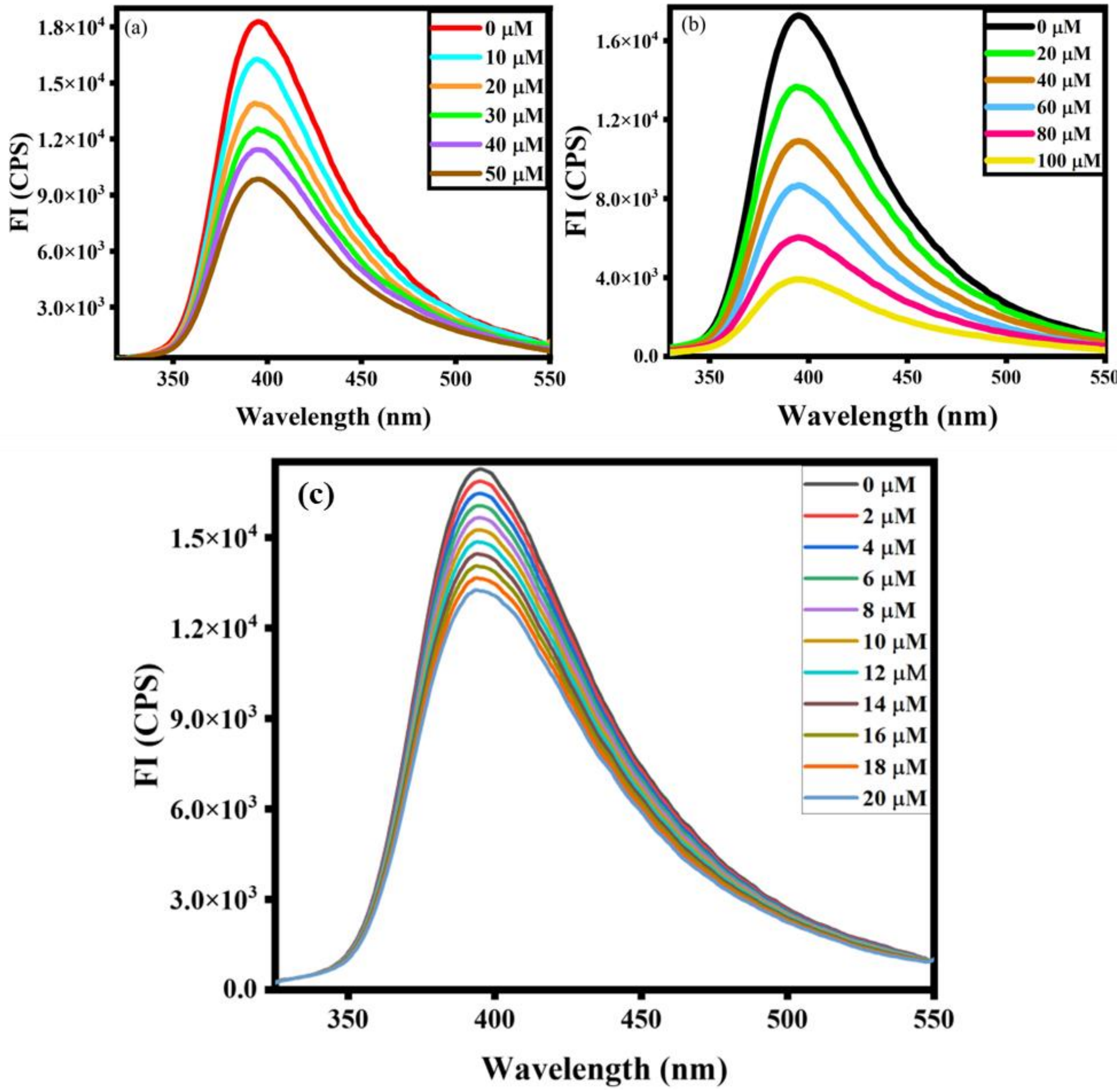

Fig. S 12. (a) Fluorescence relative emission of sensor **MPT** in plasma and (b) Real water sample (c) Fluorescence quenching observed in spiked artificial urine samples

Table. S 1 (a). The quantum yield of MPT in different water fractions and with DFX

Water Fraction	Quantum Yield	DFX Concentration(μM)	Quantum Yield
10	0.075	-	-
20	0.086	10	0.137
30	0.098	20	0.112
40	0.11	30	0.091
50	0.117	40	0.075
60	0.123	50	0.061
70	0.128	60	0.05
80	0.134	70	0.042
90	0.137	80	0.037
95	0.092	90	0.034
99	0.078	100	0.033

Table. S 1 (b). Comparison of sensor MPT with already reported DFX sensors

Sensor	LOD	Phenomenon	Reference
**Carbamothioyl based sensor**	175 nM (0.2 μM)	Fluorescence quenching	This work
**Carbon dots probe and Cu^{2+} as medium**	0.33 μg/mL (330 μM)	Fluorescence quenching	**[1]**
**Dopamine-conjugated carbon dots**	600 ng/mL (600 μM)	Fluorescence quenching	**[2]**

**Carbon dots and Cu^{2+}-catalyzed oxidation of *o*-phenylenediamine**	0.38 mg/mL (0.38 M)	Fluorescence quenching	**[3]**
**"ON-OFF-ON" sensor for sequential detection of Fe^{3+} and deferasirox**	0.14 ppm	Fluorescence enhancement	**[4]**
**Poly(Allylaminehydrochloride)-Templated Copper Nanoclusters**	0.1 µg/mL	Fluorescence quenching	**[5]**

Table. S 2. Spike and recovery experiment for detection of DFX in plasma

Spiked (µM)	Recovered (µM)	Recovery (%)	RSD (%)
10	10.4	104	1.21
20	19.7	98.5	1.18
30	30.6	102	1.26
40	41.4	103.5	1.2
50	51.8	103.6	1.29

Table. S 3. QTAIM analysis of **MPT**-DFX

BCPs	MPT-DFX	ρ(r) (a.u)	$\nabla^2\rho(r)$ (a.u)	G(r) (a.u)	V(r) (a.u)	H(r) (a.u)	-V/G	E_{int} (kcal/mol)
**1**	S…H	0.005	0.017	0.003	-0.002	-0.00106	0.68	-0.722

**2**	S... H	0.006	0.020	0.004	-0.003	0.00108	0.72	-0.878
**3**	H…C	0.004	0.011	0.002	-0.001	-0.00058	0.50	-0.314
**4**	O...H	0.025	0.076	0.020	-0.021	-0.00108	1.05	-6.589
**5**	C…H	0.006	0.021	0.004	-0.003	-0.00112	0.75	-0.941
**6**	O…O	0.010	0.046	0.010	-0.009	-0.00132	0.90	-2.823
**7**	C…N	0.004	0.012	0.002	-0.002	-0.00049	1.00	-0.628
**8**	O…H	0.028	0.094	0.023	-0.023	-0.00003	1.00	-7.216
**9**	H…O	0.013	0.042	0.010	-0.009	-0.00052	0.90	-2.824
**10**	C…O	0.009	0.028	0.006	-0.005	-0.00081	0.83	-1.569
**11**	C…N	0.004	0.011	0.002	-0.002	-0.00050	1.00	-0.628
**12**	N…H	0.006	0.020	0.004	-0.003	-0.00089	0.75	-0.941
**13**	C…H	0.007	0.021	0.004	-0.003	-0.00099	0.75	-0.941
**14**	C…C	0.006	0.019	0.004	-0.003	-0.00093	0.75	-0.941

Table. S 4. Spike and recovery values for DFX detection in real water samples

Spiked (µM)	Recovered (µM)	Recovery (%)	RSD (%)
20	19.8	99	1.24
40	40.5	101.3	1.2
60	60.9	101.5	1.32

80	81.3	101.6	1.29
100	102	102	1.17
120	121.2	101	1.19

Table S 5. Spike and recovery values for the detection of DFX in artificial urine samples

Spiked (µM)	Recovered (µM)	Recovery (%)	RSD (%)
2	1.99	99.5	0.52
4	3.96	99	0.45
6	5.87	97.8	0.4
8	7.93	99	0.35
10	9.48	94.8	0.32
12	11.79	98	0.29
14	13.92	99	0.28
16	15.89	99	0.3
18	17.9	99	0.35
20	19.73	98	0.4

References:

[1] W. Han, C. Miao, X. Zhang, Y. Lin, X. Hao, Z. Huang, S. Weng, X. Lin, X. Guo, J. Huang, A signal-off fluorescent strategy for deferasirox effective detection using carbon dots as probe and Cu^{2+} as medium, Anal. Chim. Acta, 1179 (2021) 338853, https://doi.org/10.1016/j.aca.2021.338853.

[2] C.-C. Wang, P.-T. Huang, H.-S. Kou, S.-M. Wu, Cu^{2+}-induced quenching and recovery of the luminescence of dopamine-conjugated carbon dots for sensing deferasirox in plasma, Sens. Actuators B Chem., 311 (2020) 127916, https://doi.org/10.1016/j.snb.2020.127916.

[3] C.-F. Miao, X.-Z. Guo, X.-T. Zhang, Y.-N. Lin, W.-D. Han, Z.-J. Huang, S.-H. Weng, Ratiometric fluorescence assay based on carbon dots and Cu^{2+}-catalyzed oxidation of O-phenylenediamine for the effective detection of deferasirox, RSC Adv., 11(55) (2021) 34525-34532, https://doi.org/10.1039/D1RA07078A.

[4] A.Y.A. Alzahrani, K.O. Khan, S. Rafique, H. Irshad, A.M. Khan, S.A. Shahzad, Theoretical and experimental studies on mechanochromic triphenylamine based fluorescent "ON-OFF-ON" sensor for sequential detection of Fe^{3+} and deferasirox, Spectrochim. Acta A Mol. Biomol. Spectrosc., 297 (2023) 122745, https://doi.org/10.1016/j.saa.2023.122745.

[5] H.-J. Lin, C.-C. Wang, H.-S. Kou, C.-W. Cheng, S.-M. Wu, Stable Luminescent Poly (Allylaminehydrochloride)-Templated Copper Nanoclusters for Selectively Turn-Off Sensing of Deferasirox in β-Thalassemia Plasma, Pharmaceuticals, 14(12) (2021) 1314, https://doi.org/10.3390/ph14121314.

SI-2. NMR spectra of the synthesized compound

MPT (1H-NMR, CDCl3, 400 MHz)

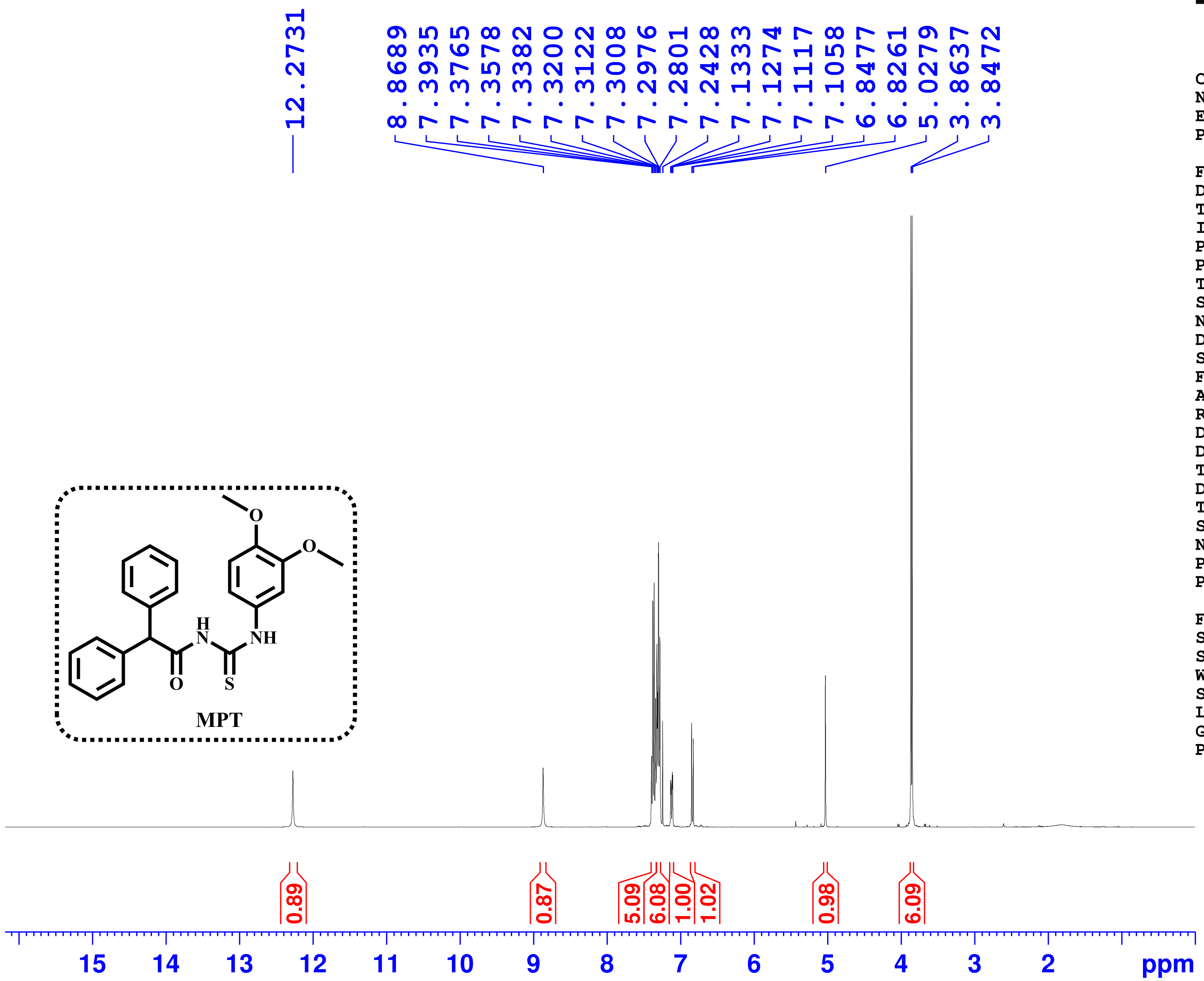

Current Data Parameters
NAME Kainat Khurshid
EXPNO 498
PROCNO 1

F2 - Acquisition Parameters
Date_ 20230831
Time 15.08 h
INSTRUM spect
PROBHD Z116098_0621 (
PULPROG zg30
TD 65536
SOLVENT CDCl3
NS 16
DS 2
SWH 8012.820 Hz
FIDRES 0.244532 Hz
AQ 4.0894465 sec
RG 88.48
DW 62.400 usec
DE 6.50 usec
TE 298.0 K
D1 1.00000000 sec
TD0 1
SFO1 400.1324708 MHz
NUC1 1H
P1 10.00 usec
PLW1 16.68099976 W

F2 - Processing parameters
SI 65536
SF 400.1300000 MHz
WDW EM
SSB 0
LB 0.30 Hz
GB 0
PC 1.00

MPT (1H-NMR, CDCl3, 400 MHz)

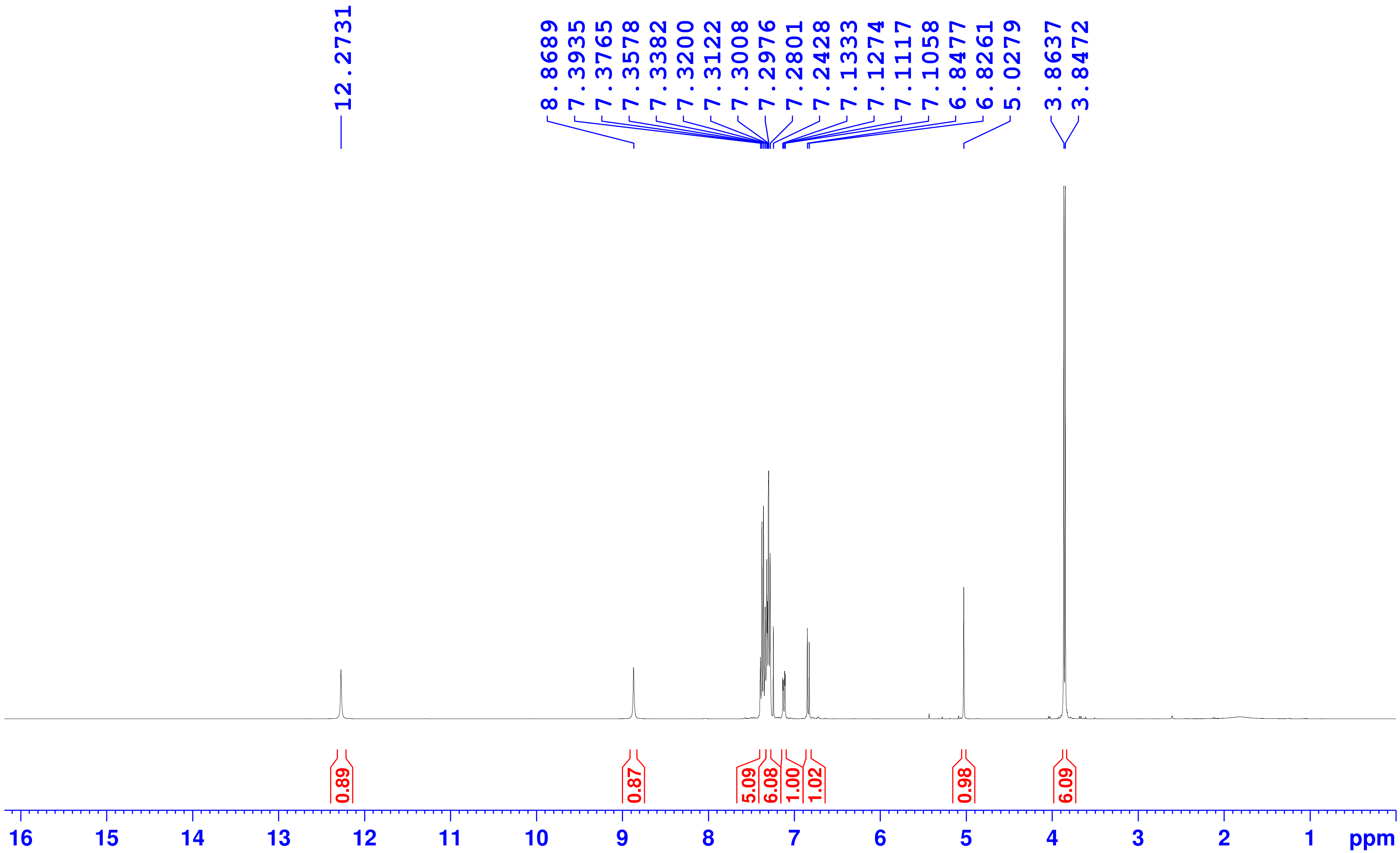

MPT (1H-NMR, CDCl3, 400 MHz)

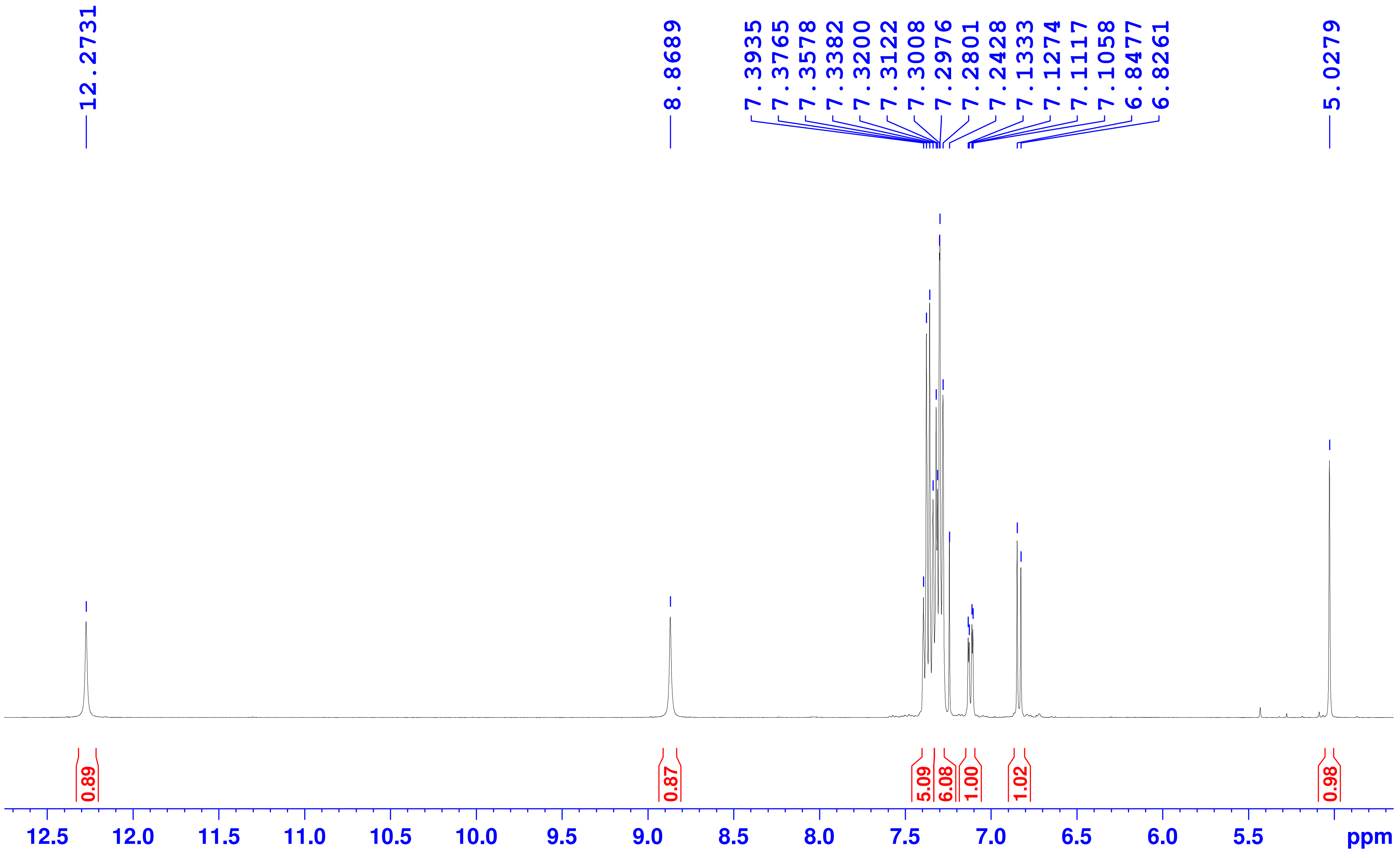

MPT (1H-NMR, CDCl3, 400 MHz)

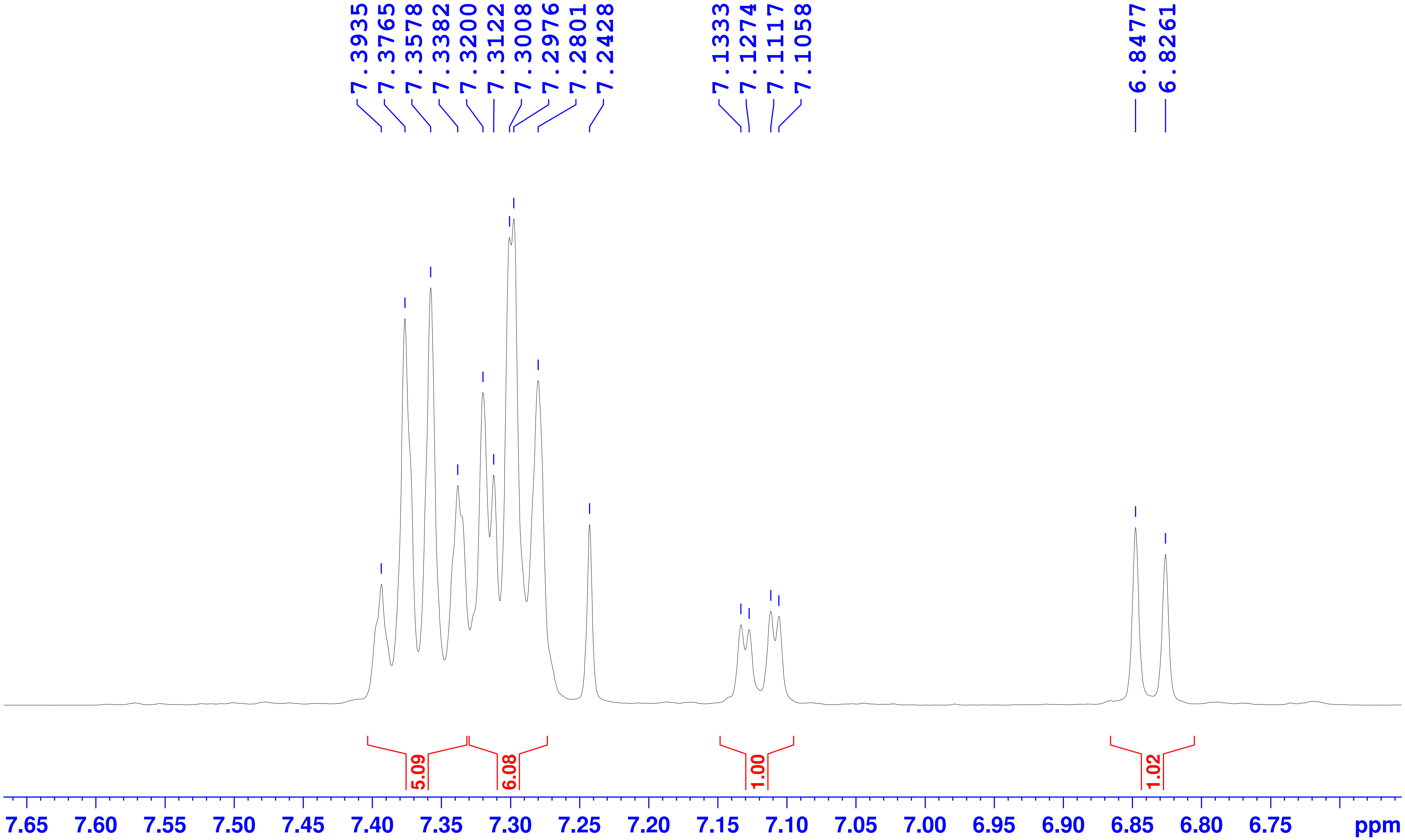

MPT (1H-NMR, CDCl3, 400 MHz)

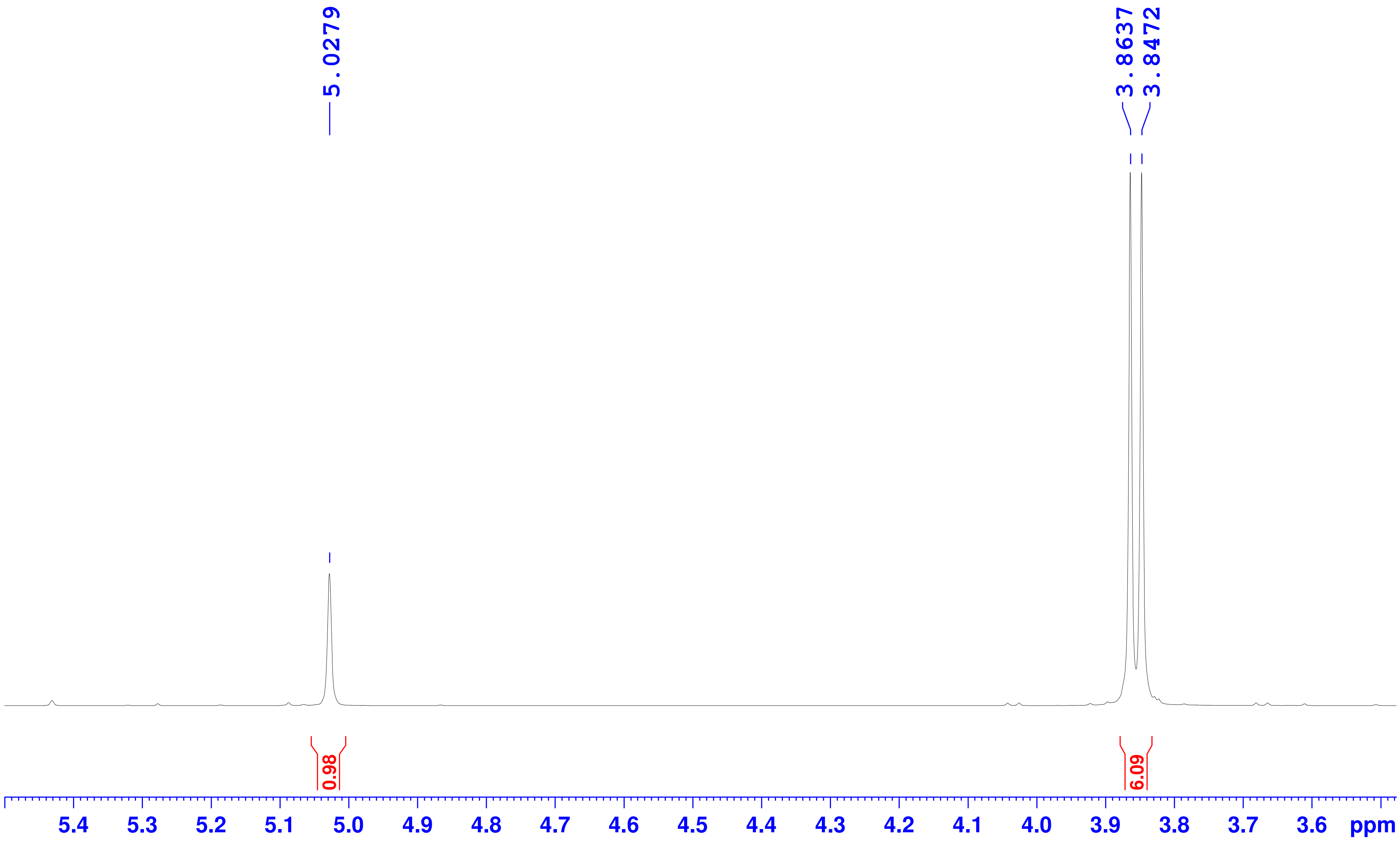

MPT (1H-NMR, CDCl3, 400 MHz)

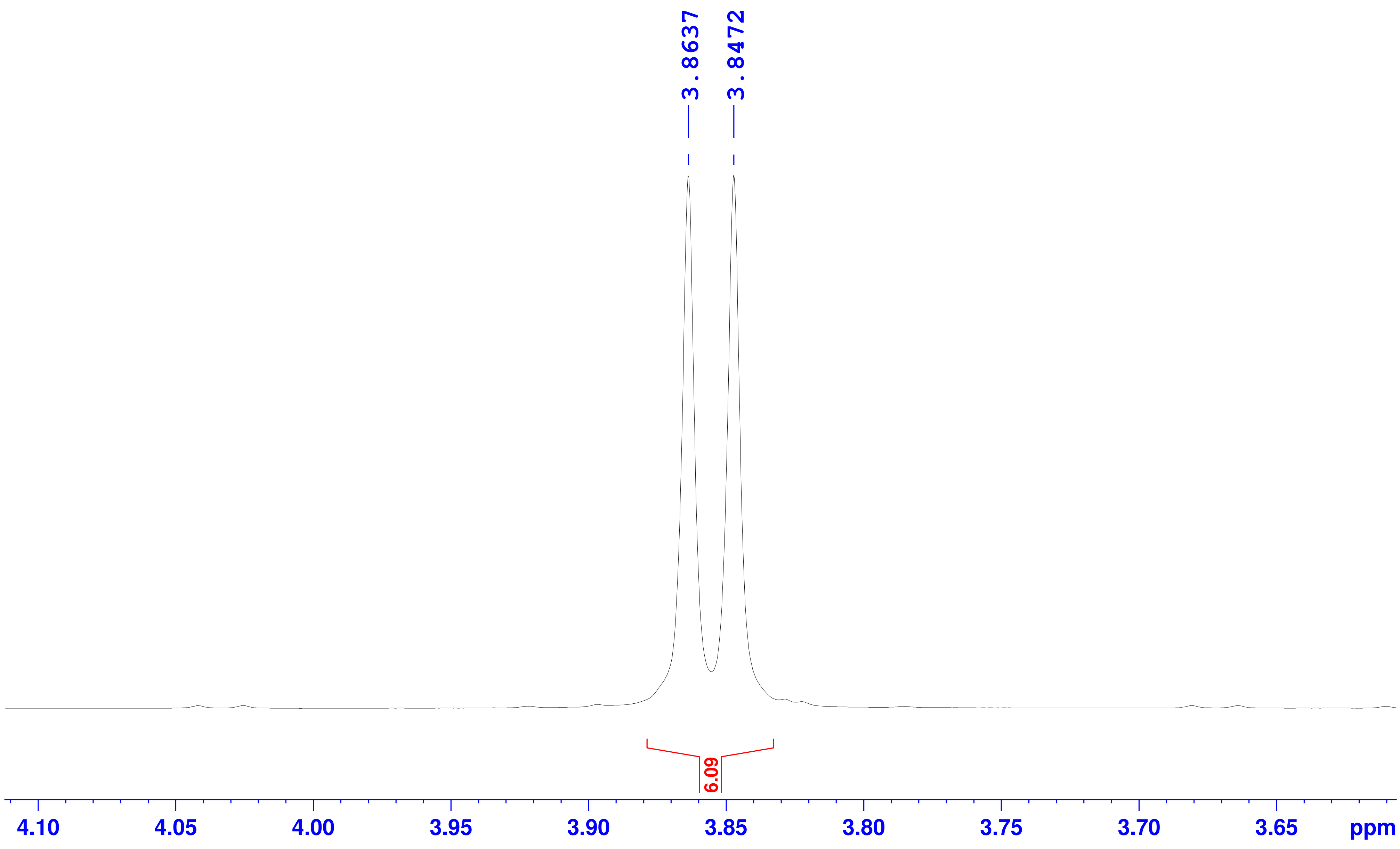

MPT (1H-NMR, CDCl3, 400 MHz)

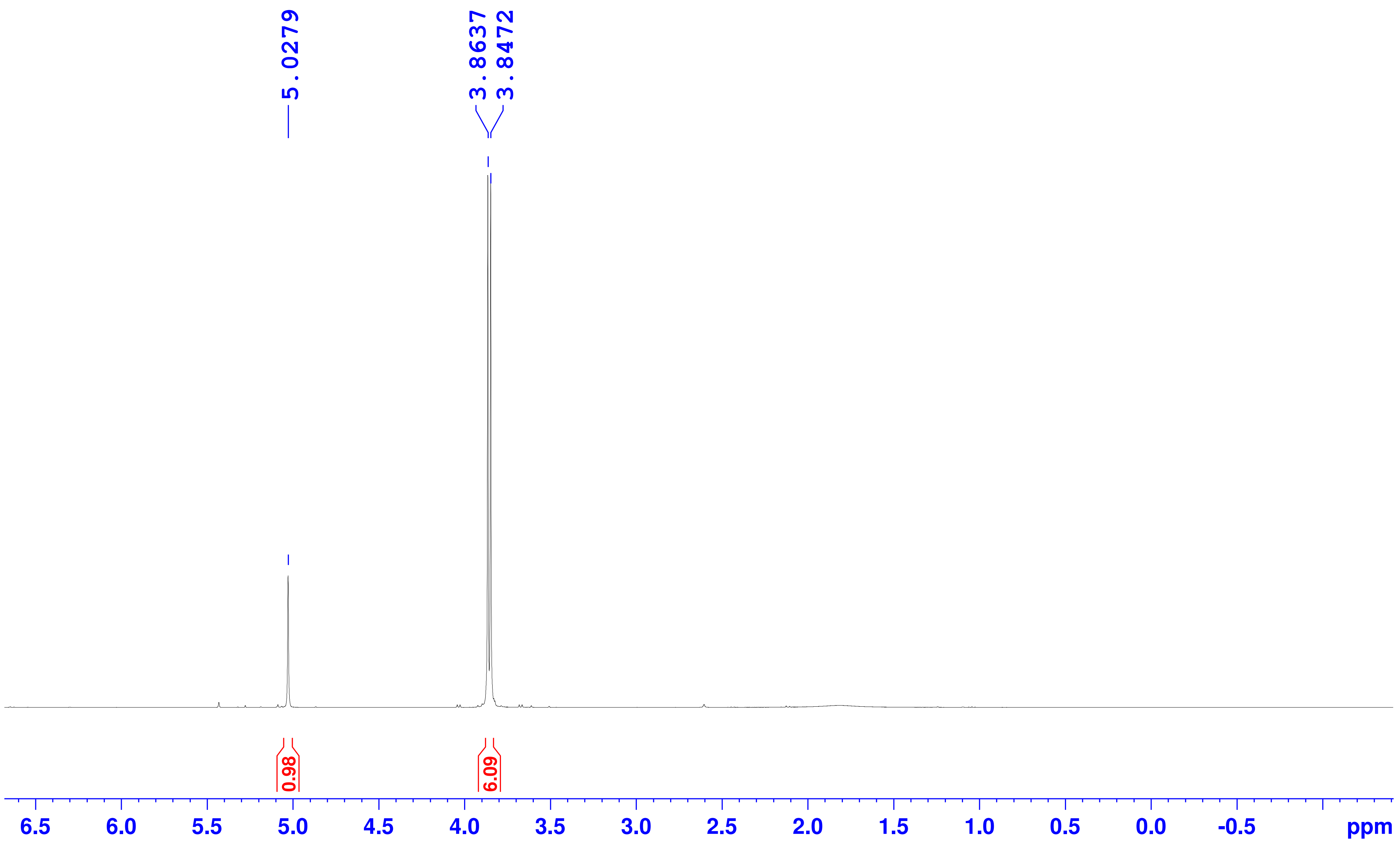

MPT (13C-NMR, CDCl3, 400 MHz)

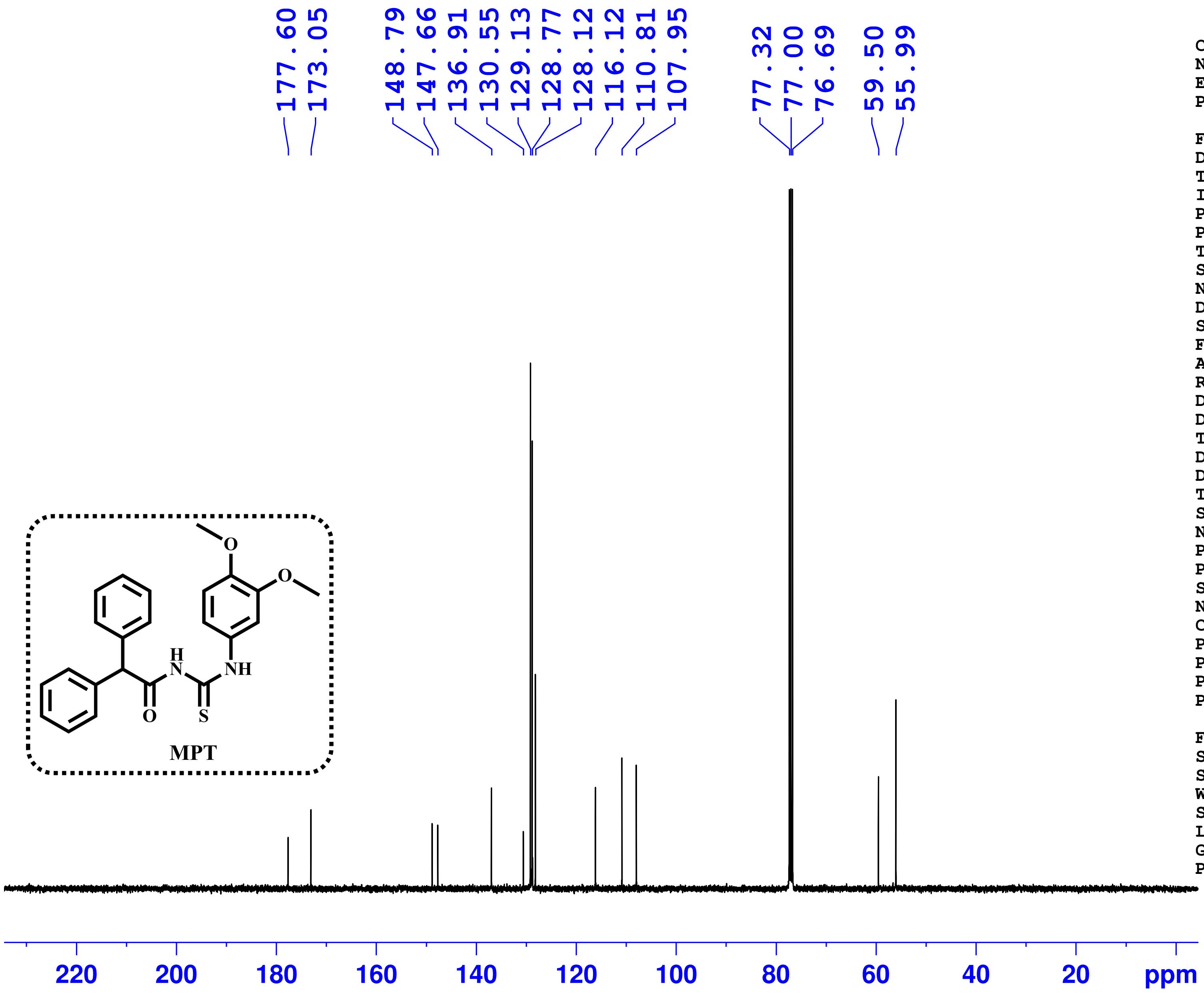

Current Data Parameters
NAME Kainat Khurshid
EXPNO 499
PROCNO 1

F2 - Acquisition Parameters
Date_ 20230901
Time 13.00 h
INSTRUM spect
PROBHD Z116098_0621 (
PULPROG zgpg30
TD 65536
SOLVENT CDCl3
NS 800
DS 2
SWH 24038.461 Hz
FIDRES 0.733596 Hz
AQ 1.3631488 sec
RG 199.48
DW 20.800 usec
DE 6.50 usec
TE 298.0 K
D1 2.00000000 sec
D11 0.03000000 sec
TD0 1
SFO1 100.6243390 MHz
NUC1 13C
P1 10.00 usec
PLW1 72.56700134 W
SFO2 400.1316005 MHz
NUC2 1H
CPDPRG[2 waltz16
PCPD2 90.00 usec
PLW2 16.68099976 W
PLW12 0.20593999 W
PLW13 0.10342000 W

F2 - Processing parameters
SI 32768
SF 100.6127685 MHz
WDW EM
SSB 0
LB 1.00 Hz
GB 0
PC 1.40

MPT (13C-NMR, CDCl3, 400 MHz)

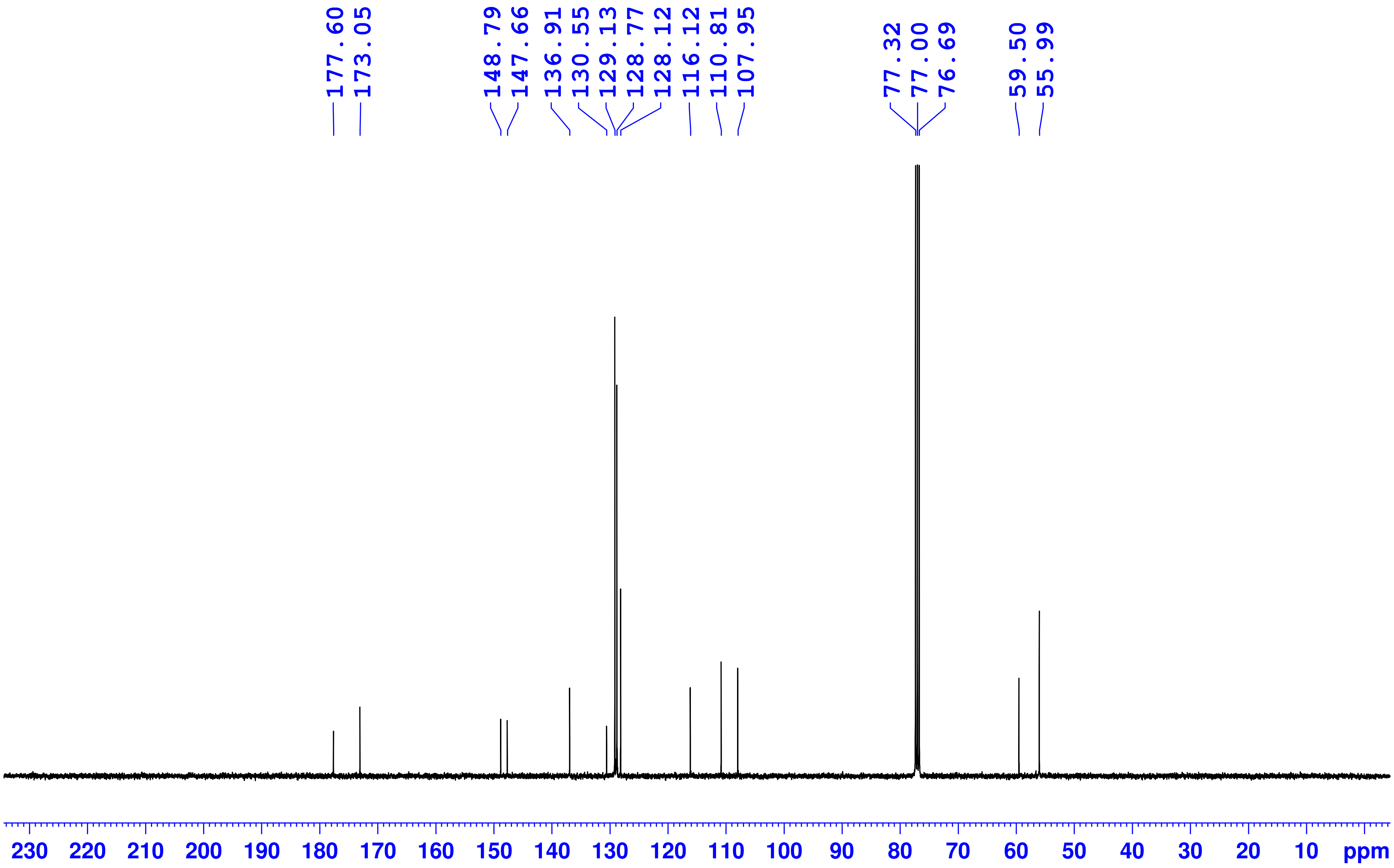

MPT (13C-NMR, CDCl3, 400 MHz)

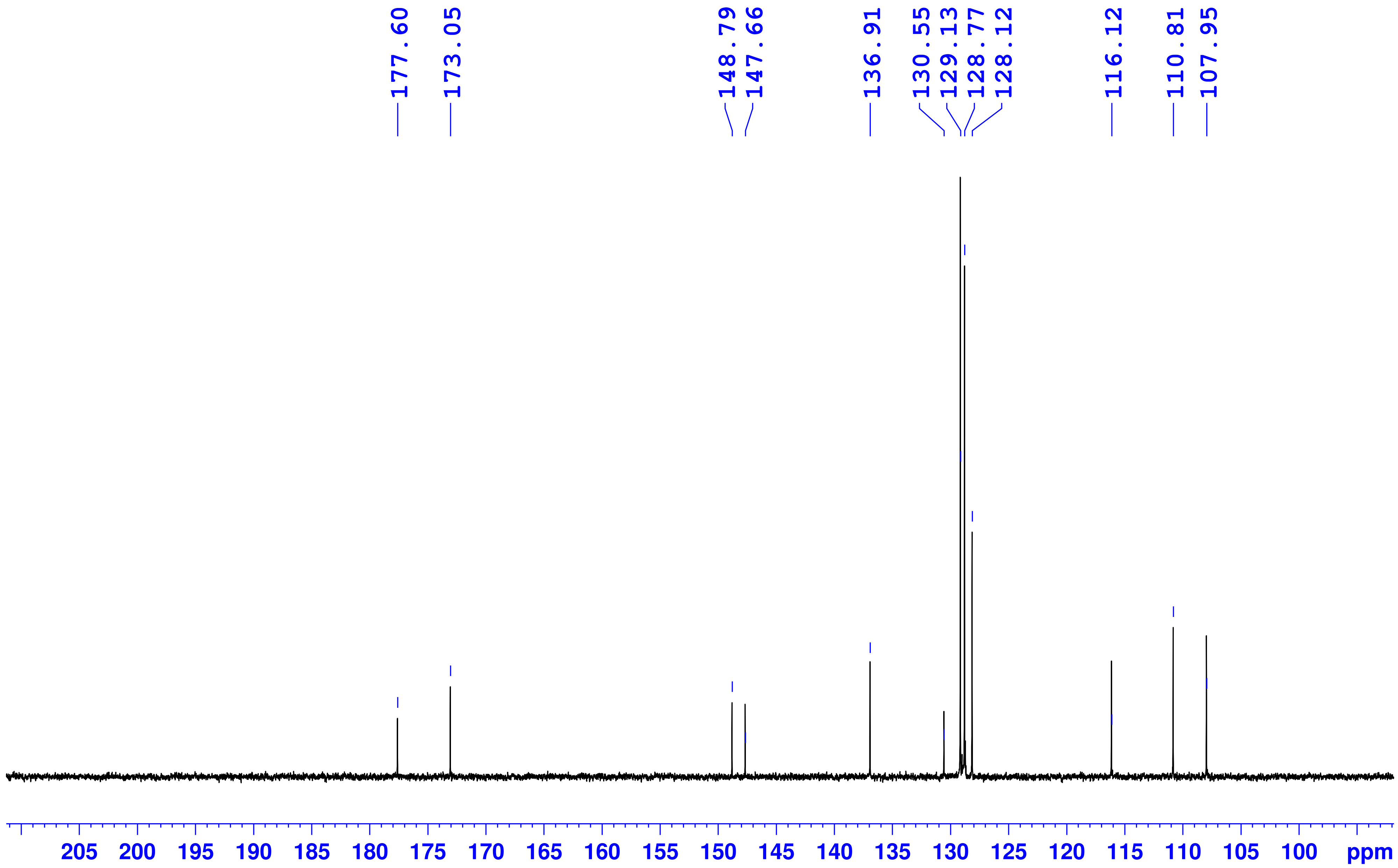

MPT (13C-NMR, CDCl3, 400 MHz)

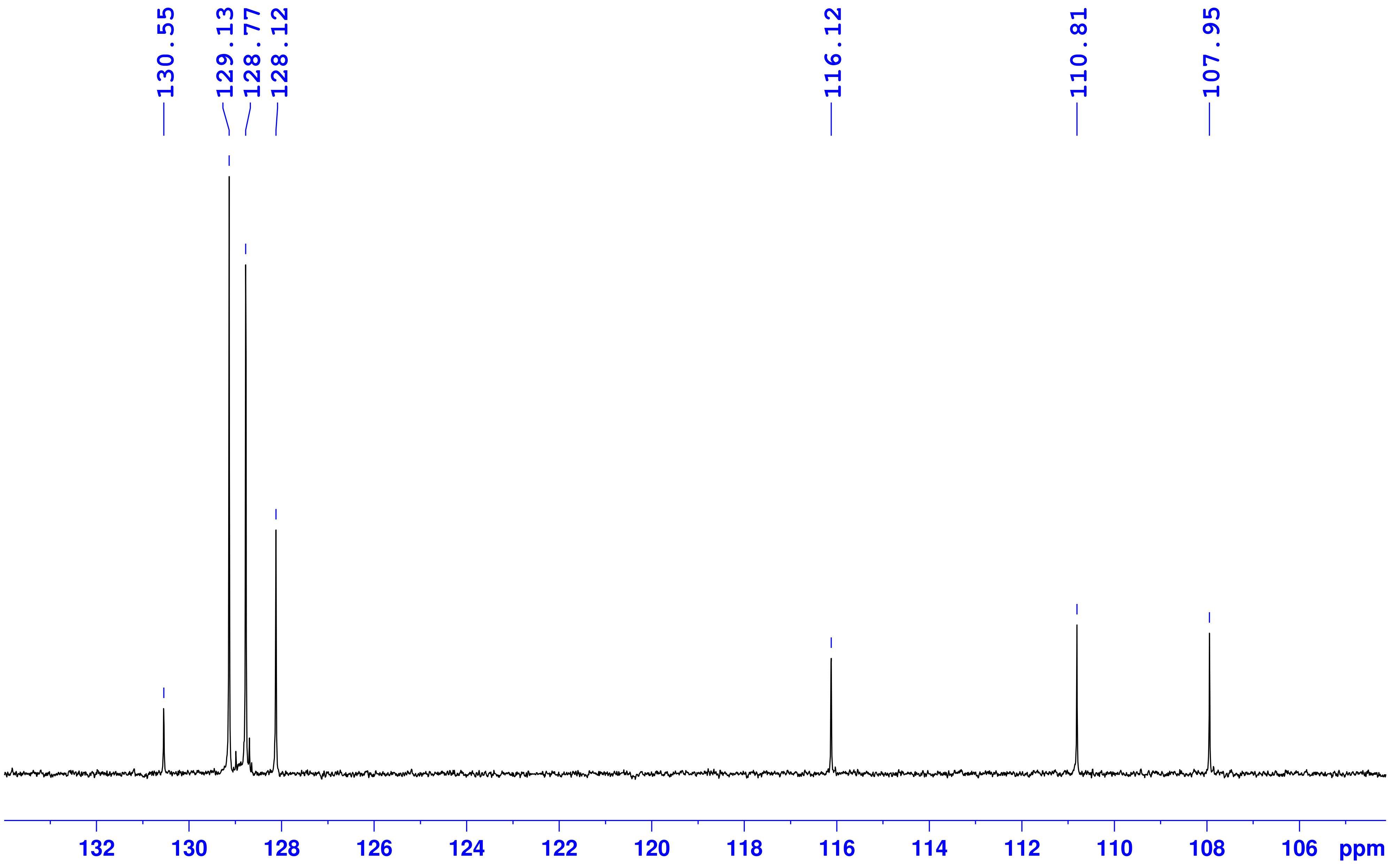

MPT (13C-NMR, CDCl3, 400 MHz)

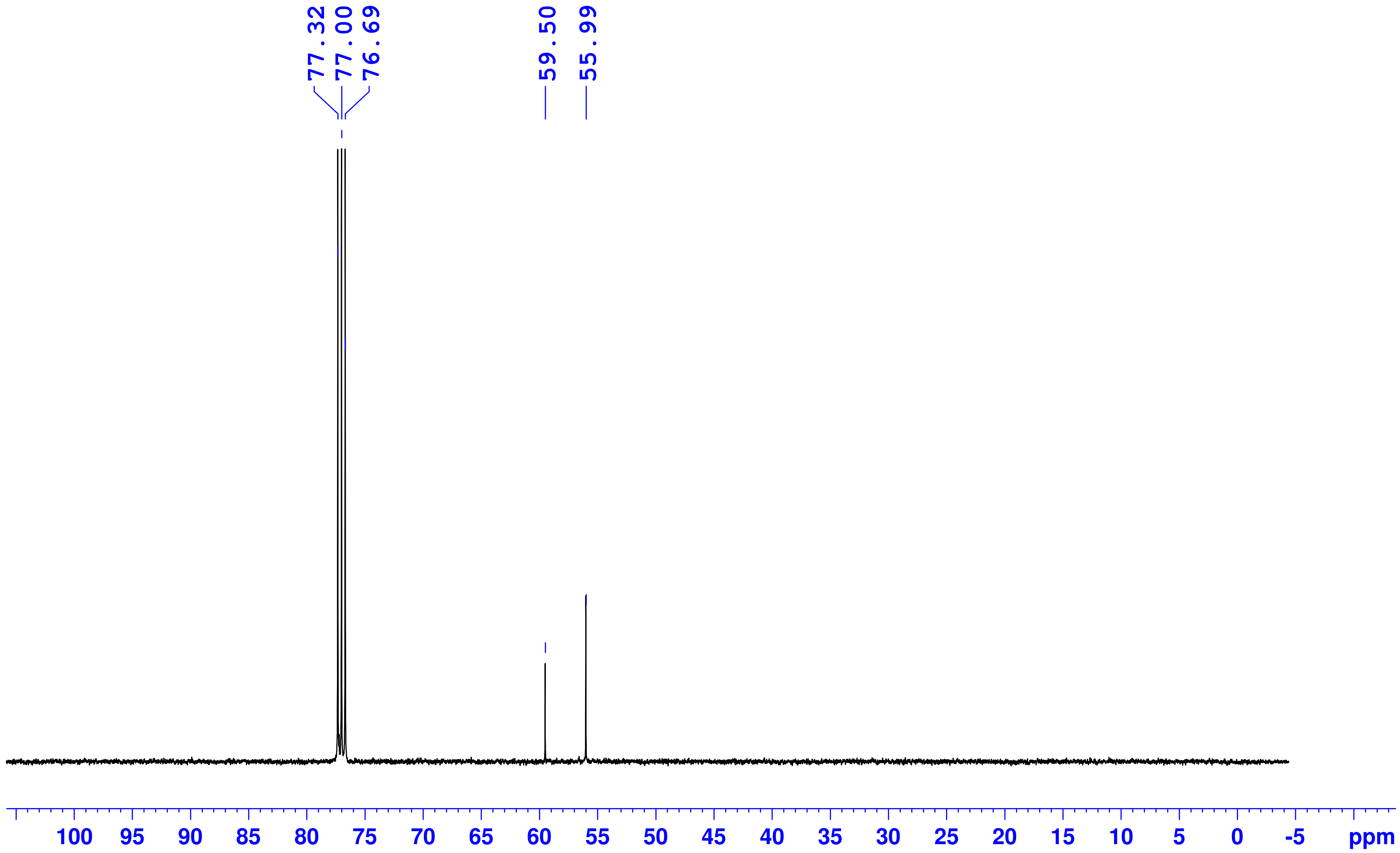

MPT (DEPT, CDCl3, 400MHz)

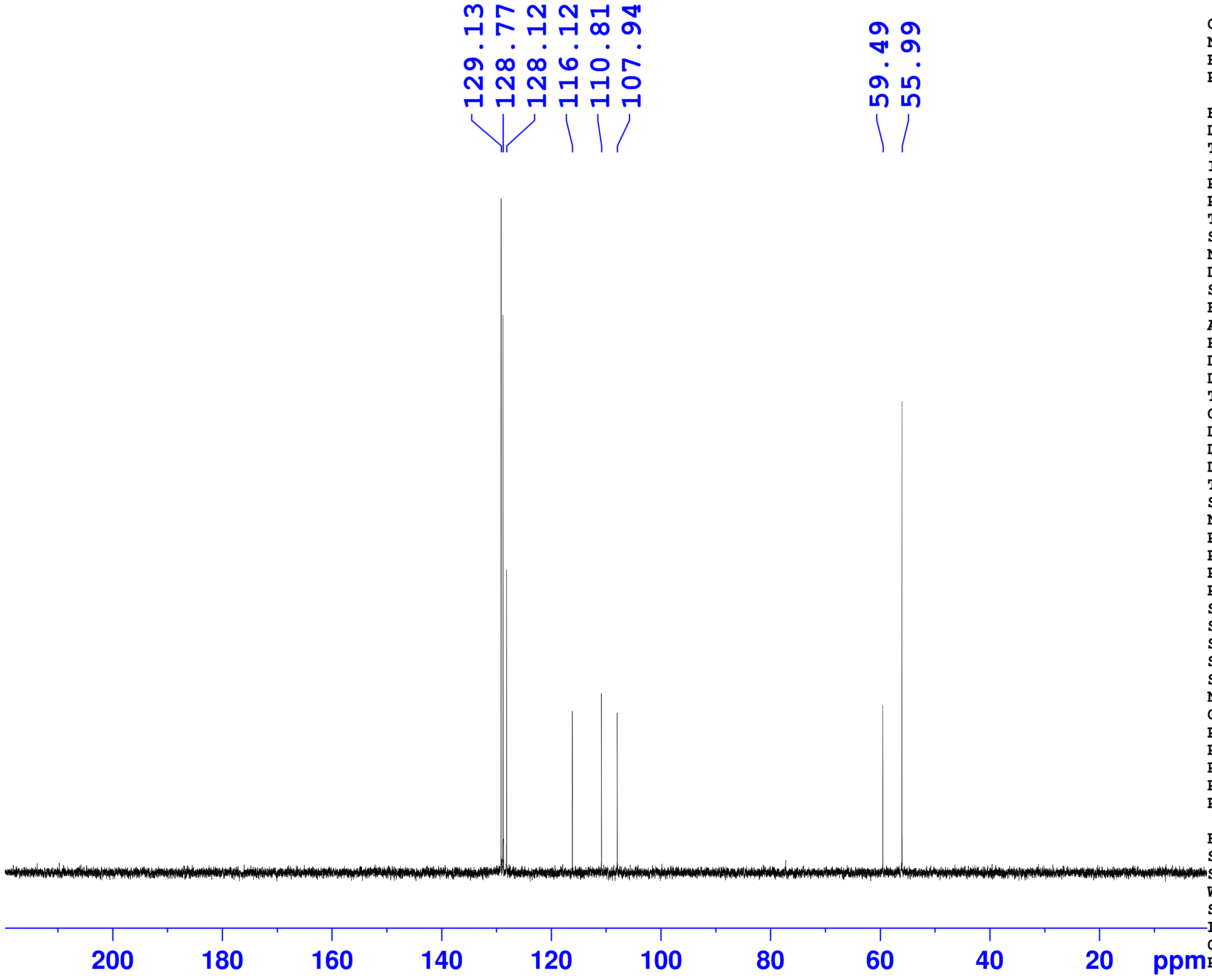

BRUKER

Current Data Parameters
NAME Kainat Khurshid
EXPNO 500
PROCNO 1

F2 - Acquisition Parameters
Date_ 20230901
Time 13.16 h
INSTRUM spect
PROBHD Z116098_0621 (
PULPROG deptsp135
TD 65536
SOLVENT CDCl3
NS 256
DS 4
SWH 22058.824 Hz
FIDRES 0.673182 Hz
AQ 1.4854827 sec
RG 199.48
DW 22.667 usec
DE 6.50 usec
TE 298.0 K
CNST2 145.0000000
D1 2.00000000 sec
D2 0.00344828 sec
D12 0.00002000 sec
TD0 1
SFO1 100.6238359 MHz
NUC1 13C
P1 10.00 usec
P13 2000.00 usec
PLW0 0 W
PLW1 72.56700134 W
SPNAM[5] Crp60comp.4
SPOAL5 0.500
SPOFFS5 0 Hz
SPW5 11.08699989 W
SFO2 400.1316005 MHz
NUC2 1H
CPDPRG[2 waltz16
P3 10.00 usec
P4 20.00 usec
PCPD2 90.00 usec
PLW2 16.68099976 W
PLW12 0.20593999 W

F2 - Processing parameters
SI 32768
SF 100.6127685 MHz
WDW EM
SSB 0
LB 1.00 Hz
GB 0
PC 1.40

MPT (DEPT, CDCl3, 400MHz)

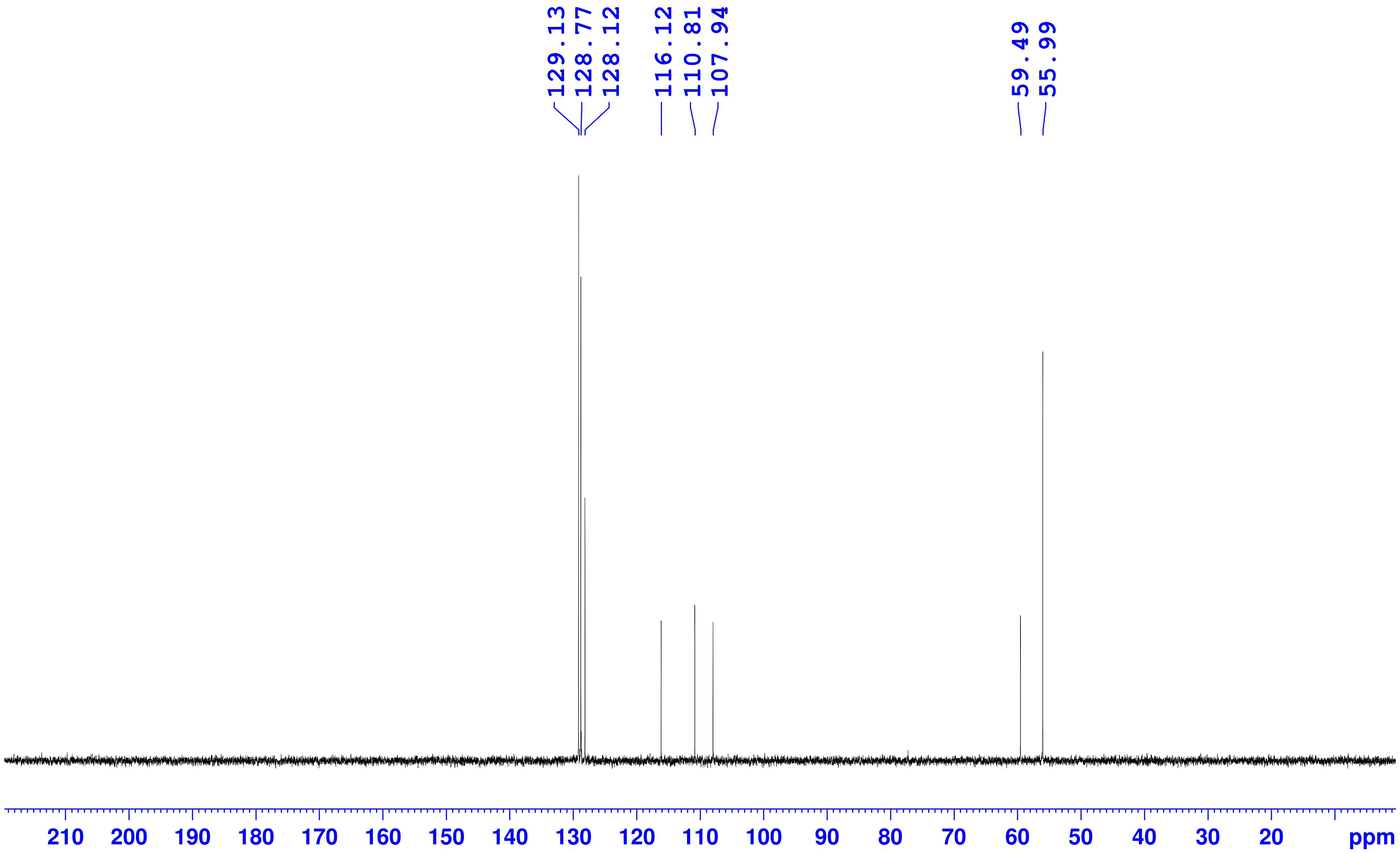

MPT (DEPT, CDCl3, 400MHz)

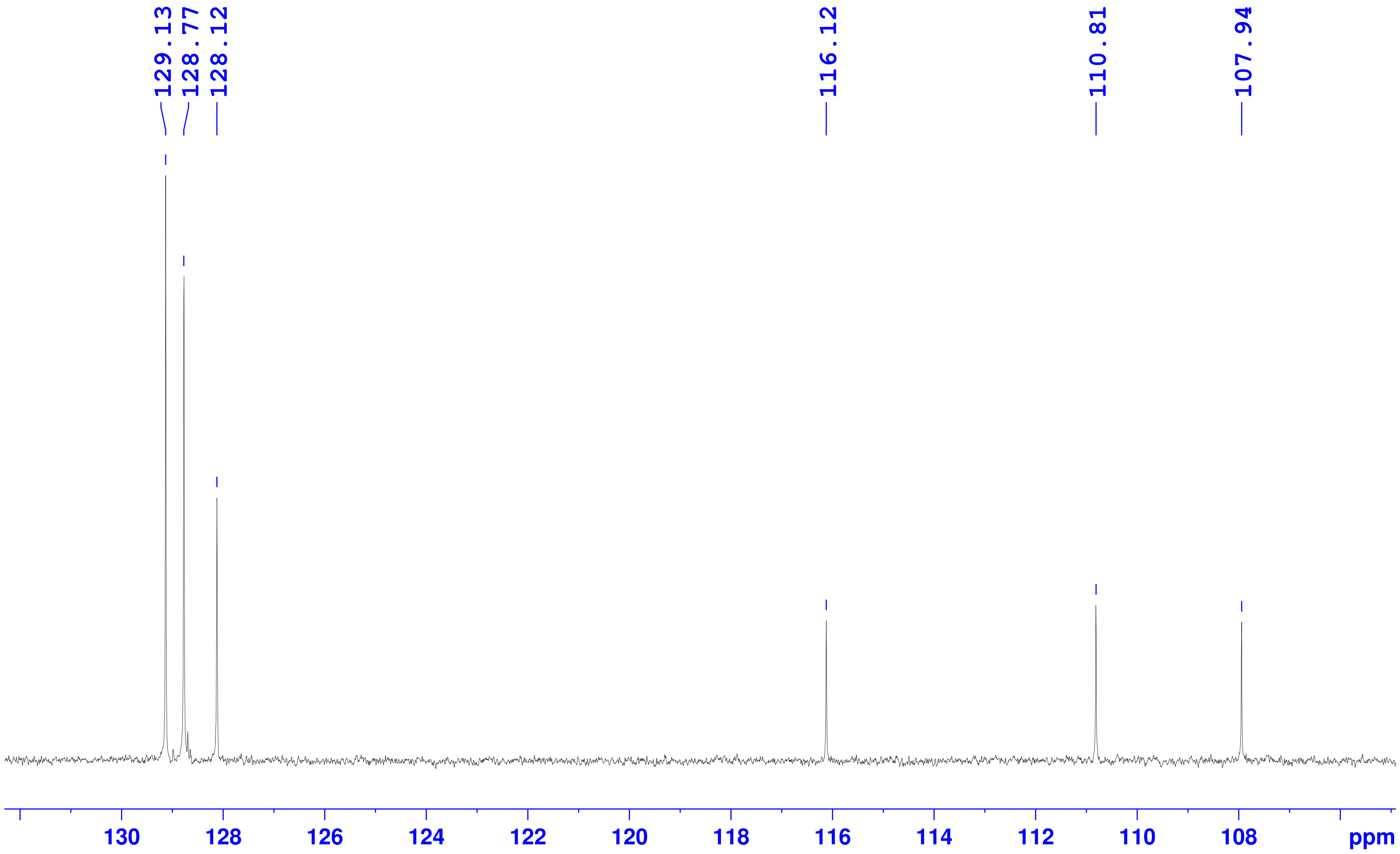

MPT (DEPT, CDCl3, 400MHz)

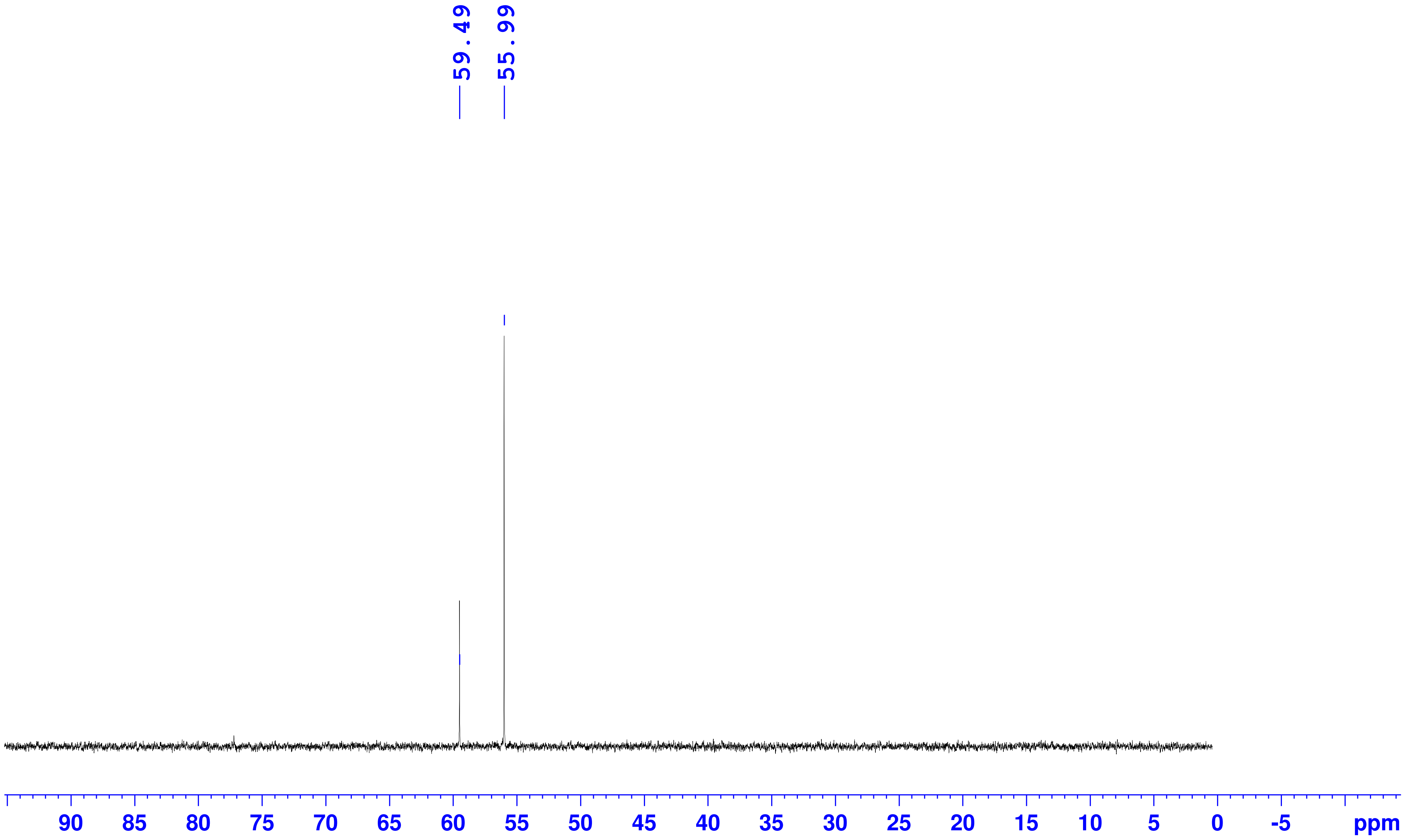

Supplement: RA-014-D4RA03548H-s001 [file RA-014-D4RA03548H-s001.pdf]
